# Trans-dimensional Bayesian model averaging for ^13^C-metabolic flux analysis: evidence-based flux inference under structural model uncertainty

**DOI:** 10.1093/bioinformatics/btag587

**Published:** 2026-08-04

**Authors:** Johann F Jadebeck, Anton Stratmann, Martin Beyß, Katharina Nöh

**Affiliations:** Institute of Bio- and Geosciences, Forschungszentrum Jülich, Wilhelm-Johnen Straße, Jülich 52428, Germany; Computational Systems Biotechnology (AVT.CSB), RWTH Aachen University, Aachen 52074, Germany; Institute of Bio- and Geosciences, Forschungszentrum Jülich, Wilhelm-Johnen Straße, Jülich 52428, Germany; Computational Systems Biotechnology (AVT.CSB), RWTH Aachen University, Aachen 52074, Germany; Institute of Bio- and Geosciences, Forschungszentrum Jülich, Wilhelm-Johnen Straße, Jülich 52428, Germany; Institute of Bio- and Geosciences, Forschungszentrum Jülich, Wilhelm-Johnen Straße, Jülich 52428, Germany

## Abstract

**Motivation:**

Accurate quantification of intracellular metabolic fluxes is central to systems biology and biotechnology. Flux estimation relies on biochemical network models, with ^13^C-metabolic flux analysis (MFA) being the state-of-the-art approach. However, isotope labeling data are often insufficient to uniquely support a single network formulation. In such cases, flux estimates become model-dependent, highlighting the need for methods that explicitly account for structural uncertainty. Bayesian model averaging provides a principled framework for this purpose, but its application to ^13^C-MFA has so far been restricted to uncertainty in reaction bidirectionality within fixed network topologies.

**Results:**

We introduce a scalable Bayesian inference framework for ^13^C-MFA, Bayesian model set averaging, that applies Bayesian model averaging to encompass uncertainty in reactions and pathways. Our approach combines reversible jump Markov chain Monte Carlo for trans-dimensional exploration of structural hypotheses with diffusive nested sampling for robust estimation of model evidences, enabling averaging over large families of metabolic network structures. Using illustrative and application-scale synthetic case studies, we demonstrate that the method yields robust flux estimates, reveals when multiple network configurations are statistically indistinguishable, and recovers the correct data-supported pathway configuration as data informativeness increases. Rather than conditioning inference on a single assumed network structure, the framework quantifies posterior support for alternative reaction and pathway hypotheses and propagates the resulting structural uncertainty into flux estimates. The approach scales to billions of model variants, providing a practical foundation for quantitative Bayesian flux inference under structural model uncertainty in ^13^C-MFA.

**Availability and implementation:**

Sources and scripts to replicate results are available at https://github.com/JuBiotech/Supplement-to-Jadebeck-et-al.-2026.

## 1 Introduction

Metabolism plays a central role in systems biology, biotechnology, and metabolic engineering. Quantitative understanding of intracellular metabolic processes is essential for the design of sustainable bioprocesses (Liu *et al.* 2025), for dissecting host-pathogen interactions and drug responses (Beste *et al.* 2013), and for uncovering metabolic reprogramming in cancer (Antoniewicz 2018). Decades of biochemical research and genomics, complemented by computational platforms (Gong *et al.* 2024), have yielded comprehensive reconstructions of the biochemical reactions that *can* occur in a cell. However, determining which of these reactions are active under a given physiological state, and at what rates, requires condition-specific data. Reaction activity depends strongly on physiological state and environmental context: e.g. latent metabolic pathways may only become active under genetic or environmental perturbations (Fong *et al.* 2006).

Metabolic fluxes represent the integrated functional outcome of cellular processes and are therefore central to biological interpretation (Sauer 2006). However, fluxes are not directly observable and must be inferred from data using mechanistic models. Isotope-based metabolic flux analysis (^13^C-MFA) is a widely used technology for estimating intracellular fluxes (Zamboni *et al.* 2009, Niedenführ *et al.* 2015, Long and Antoniewicz 2019). It combines isotope-labeling patterns and extracellular exchange rates collected under metabolic (pseudo)steady-state conditions with a metabolic model to solve an inverse problem. This inverse problem is typically ill-posed, meaning that the available measurements often do not uniquely determine all intracellular fluxes (Wiechert and Nöh 2021). Advances in the automation of isotope-labeling experiments (ILE) and the integrative analysis of multiple datasets using different tracers improve precision (Leighty and Antoniewicz 2013). Nevertheless, many fluxes remain non-identifiable due to limited data informativeness and structural non-identifiability inherent to metabolic cycles (Kappelmann *et al.* 2016).

A fundamental challenge in ^13^C-MFA is its dependence on the assumed metabolic network model. The model must capture the relevant metabolic pathways active under the studied experimental condition (Long and Antoniewicz 2019, Theorell *et al.* 2024). Simply including all known reactions is not a solution: large networks introduce alternative routes that cannot be discriminated by the available data, increasing non-identifiability and the risk of biologically implausible solutions (Linden-Santangeli and Rangamani 2025). Moreover, the increased number of candidate models and flux parameters makes inference computationally more demanding, particularly when weakly informative data provide limited posterior concentration. Evidence-based model averaging addresses this issue by favoring additional reaction and pathway hypotheses only when the increased model flexibility is supported by sufficient statistical evidence in the data. In practice, carefully curated core models are therefore commonly employed to maintain identifiability. Yet, structural uncertainty remains even within such models. In particular, the presence of latent pathways can lead to substantially different flux estimates depending on the chosen model formulation (Fong *et al.* 2006, Zamboni *et al.* 2009), underlining that flux estimation is inherently model-dependent.

In practice, this dependency gives rise to a model selection problem: identifying which model(s) are supported by the data. Standard approaches based on goodness-of-fit are insufficient for reliable model discrimination (Sundqvist *et al.* 2022). Information criteria such as Akaike (AIC) or Bayesian (BIC) account for model complexity but become difficult to apply in combinatorial model spaces (Theorell *et al.* 2024). Validation-based approaches using held-out data (Sundqvist *et al.* 2022) provide an alternative, but require sufficiently comprehensive datasets, limiting their applicability when data are scarce or redundant.

We here present an alternative approach that avoids committing to a single model. We adopt a Bayesian framework to explicitly account for structural model uncertainty at the level of reactions and pathways. Using Bayesian model averaging (BMA; Hoeting *et al.* 1999), we treat network structure (the set of reactions that constitutes the model) as a variable and propagate both data and structural uncertainty into flux estimates by averaging over a family of candidate models. The candidate model set is constructed from biochemical knowledge and assumed to be sufficiently comprehensive to capture the underlying metabolic processes.

Instead of conditioning inference on a single model, BMA yields flux estimates that reflect the support of all alternative model hypotheses. By weighting models according to their posterior probability, the approach quantifies relative support within the model space and reduces overconfidence associated with single-model inference. Posterior model probabilities quantify statistical support within the specified candidate model family rather than claims of model correctness.

In this work, we generalize the approach of Theorell *et al.* (2024), who considered uncertainty in reaction bidirectionality within a fixed network, to also account for uncertainty in reactions and pathways. The resulting inference problem is computationally challenging, combining nonlinear, potentially multimodal likelihoods with linearly constrained flux spaces that vary across models. To address this, we develop a scalable inference algorithm that combines reversible jump Markov chain Monte Carlo (RJMCMC) for exploring models with different numbers of flux parameters (i.e. trans-dimensional exploration of the model space) with diffusive nested sampling (DNS) for robust and interpretable model evidence estimation (Green 1995, Brewer *et al.* 2011). We evaluate our approach using simulated data, focusing on flux inference under structural uncertainty for both illustrative and application-scale ^13^C-MFA scenarios. Using known ground truth, we demonstrate both computational feasibility and how evidence-based model averaging should be interpreted under different levels of data informativeness.

## 2 Problem statement

We first revisit Bayesian flux inference under a single model and under uncertain reaction bidirectionality, before generalizing the formulation to uncertainty in reactions and pathways.

### 2.1 Bayesian single-model metabolic flux inference

Given an ILE dataset D, the conventional Bayesian approach uses a single network model M and infers the posterior probability density $p(v_{M}|D)$ of the model-specific fluxes $v_{M}$ (Theorell *et al.* 2017). The subscript emphasizes that different models have different model-specific flux vectors and parameter spaces. The model M consists of a set of relevant biochemical reactions, which we refer to as *reaction set*, associated atom transitions, and assumed reaction bidirectionality (Wiechert and de Graaf 1997). Bidirectional reactions are parametrized by net and exchange fluxes, where the exchange flux quantifies the simultaneous forward and reverse turnover of the reaction. This should not be confused with extracellular fluxes across the cell boundary. Unidirectional reactions contribute only net fluxes, while their exchange fluxes are zero.

Under metabolic (pseudo)steady-state conditions, the reaction stoichiometry of M together with physiological flux limits induces linear mass balance constraints that restrict fluxes to a bounded, convex polytope $P_{M}$ (Theorell *et al.* 2022). Its dimension, i.e. the number of independent (free) fluxes, defines the degrees of freedom (DOF) of the inference problem. The flux posterior $p(v_{M}|D)$ is typically explored using Markov chain Monte Carlo (MCMC) (Theorell *et al.* 2017).

### 2.2 Flux inference at uncertain reaction bidirectionality

A key limitation of single-model ^13^C-MFA is the assumption that the model structure is correct. In particular, reaction bidirectionality depends on *in vivo* thermodynamic driving forces that are not precisely known (Wiechert 2007), and exchange fluxes are typically weakly identifiable (Wiechert *et al.* 1997). Consequently, uncertainty in reaction bidirectionality gives rise to many model variants with different DOF. Specifically, for $n_{x}$ reactions with uncertain bidirectionality, $2^{n_{x}}$ unique model variants exist. Typical ^13^C-MFA models therefore comprise from a few dozen to several hundred thousand model variants (Theorell *et al.* 2024).

To account for this uncertainty, the finite set ${\left\{M\right\}}_{I}$ of candidate models is considered and BMA is applied, which combines the single-model flux posteriors $p(v_{M_{i}}|D)$ with the associated posterior model probabilities $p(M_{i}|{\left\{M\right\}}_{I},D)$ (where I denotes the index set of candidate models) conditioned on the model set in a weighted average to determine model set-averaged fluxes $v_{{\left\{M\right\}}_{I}}$


(1) $$p(v_{{\left\{M\right\}}_{I}}|D)=\sum_{i^{\prime }\in I}p(M_{i^{\prime }}|{\left\{M\right\}}_{I},D)\cdot p(v_{M_{i^{\prime }}}|D)$$

where model set-averaged fluxes are defined on the shared flux space $P_{{\left\{M\right\}}_{I}}$, by assigning zero values to parameters absent in a given model. Posterior model probabilities $p(M_{i}|{\left\{M\right\}}_{I},D)$ represent the relative support of alternative model structures given D. Structural uncertainty is treated explicitly through alternative model structures rather than continuous flux uncertainty because different reaction sets define different parameter spaces.

Direct evaluation of all posterior model probabilities in Equation (1) is computationally prohibitive, as it requires high-dimensional integration over convex polytopes. RJMCMC provides a practical alternative by jointly sampling (discrete) model structures and continuous fluxes, thereby avoiding explicit enumeration of millions of model evidences (Green 1995, Theorell and Nöh 2020). This approach has been shown to improve the robustness of flux inference (Theorell *et al.* 2024) and is applied in ^13^C-MFA (Borah Slater *et al.* 2023).

### 2.3 Flux inference at full model uncertainty

While the approach by Theorell and Nöh (2020) accounts for uncertainty in reaction bidirectionality, it still assumes that the underlying reaction set is correct. This assumption is often questionable, particularly in the presence of latent pathways whose activity, i.e. whether a pathway carries a non-zero net flux, is condition-dependent (Zamboni *et al.* 2009).

We extend Equation (1) to account for uncertainty at the level of both reaction sets (reactions and pathways) and reaction bidirectionality. Let $M^{k}$, k=1…K denote *K* alternative reaction sets, each containing $n_{x,k}$ reactions with uncertain bidirectionality. Each reaction set induces a model set ${\left\{M^{k}\right\}}_{I^{k}}$ with $2^{n_{x,k}}$ variants. The full candidate model space is then given by


(2) $${\left\{M^{k}\right\}}_{K}=\overset{K}{\underset{k^{\prime }=1}{\cup }}{\left\{M^{k^{\prime }}\right\}}_{I^{k^{\prime }}}\;\text{with}\;K=\overset{K}{\underset{k^{\prime }=1}{\cup }}I^{k^{\prime }}$$

containing $\sum_{k^{\prime }=1}^{K}2^{n_{x,k^{\prime }}}$ model variants.

The BMA formulation extends naturally to this setting: the model set-averaged flux posterior given the full model set is


(3) $$p(v_{{\left\{M^{k}\right\}}_{K}}|D)=\sum_{k^{\prime }=1,\;i^{\prime }\in I^{k^{\prime }}}^{K}p(M_{i^{\prime }}^{k^{\prime }}|{\left\{M^{k^{\prime }}\right\}}_{K},D)\cdot p(v_{M_{i^{\prime }}^{k^{\prime }}}|D)$$

with the single-model posterior probabilities for any candidate model $M_{i^{\prime }}^{k^{\prime }}$ in view of the full model set is given


(4) $$\begin{array}{c} p(M_{i^{\prime }}^{k^{\prime }}|{\left\{M^{k^{\prime }}\right\}}_{K},D)=\frac{p(D|M_{i^{\prime }}^{k^{\prime }})\cdot p(M_{i^{\prime }}^{k^{\prime }})}{\sum_{k^{\prime \prime }=1,i^{\prime \prime }\in I^{k^{\prime \prime }}}^{K}\;p(D|M_{i^{\prime \prime }}^{k^{\prime \prime }})\cdot p(M_{i^{\prime \prime }}^{k^{\prime \prime }})} \end{array}$$

with model priors $p(M_{i}^{k})$. Herein, a key quantity is the model evidence


(5) $$p(D|M_{i}^{k})=\int_{P_{M_{i}^{k}}}p(D|v_{M_{i}^{k}})\cdot p(v_{M_{i}^{k}}|M_{i}^{k})\;dv$$

with the model-specific likelihood $p(D|v_{M_{i}^{k}})$, which quantifies the fit quality of the model $M_{i}^{k}$ with respect to the data D, and the flux prior $p(v_{M_{i}^{k}}|M_{i}^{k})$. The evidence therefore measures the average ability of a model to explain the data over its feasible flux polytope, naturally balancing fit quality against flexibility. Evaluating the integral in Equation (5) across combinatorial model spaces constitutes the main computational challenge we address in this work.

### 2.4 A note on priors in ^13^C-MFA

The Bayesian formulation in Equations (4) and (5) requires priors over both model structures and flux parameters. As in all Bayesian model selection frameworks, model probabilities and evidences may be sensitive to prior choices (Kass and Raftery 1995), making careful prior specification an important aspect of practical applications. Candidate reaction sets are typically derived from biochemical knowledge bases such as KEGG (https://www.genome.jp/kegg/) and BioCyc (http://biocyc.org/), but these resources do not resolve condition-specific reaction or pathway activity, nor reaction bidirectionality (Fong *et al.* 2006, Nishikawa *et al.* 2008).

We adopt the following pragmatic choices. First, we assume that the candidate model space is sufficiently comprehensive to capture the relevant metabolic processes, while excluding implausible variants (near M-closed setting). Consequently, posterior model probabilities are interpreted as relative support within this space. Second, to avoid bias toward reaction sets that induce more model variants (due to a larger number of reactions with uncertain bidirectionality), we assign equal prior mass to each reaction set and uniform model priors within each set, yielding $p(M_{i}^{k}|{\left\{M^{k}\right\}}_{K})=1/(K\cdot 2^{n_{x,k}})$. Third, for the single-model fluxes we use uniform priors over the respective flux polytope $P_{M_{i}^{k}}$. Because the flux polytope encodes stoichiometric mass balances at metabolic steady state, reaction directionality, bidirectionality, assumptions and flux bounds, this prior is proper by construction and weakly informative rather than uninformative (Theorell *et al.* 2024). More generally, prior information on reaction/pathway activity (e.g. from omics-supported context-specific network reconstruction), reaction bidirectionality, or flux bounds should and can be incorporated whenever available. Such information is easily encoded as informative priors or used to restrict the candidate model family, thereby improving inference quality.

## 3 A divide-and-conquer approach to flux inference

Directly applying RJMCMC to infer model-averaged fluxes under full model uncertainty is difficult because each model is associated with a different flux polytope and a nonlinear likelihood. Although efficient samplers exist for unimodal distributions defined on convex domains, ^13^C-MFA posteriors frequently exhibit strong nonlinear parameter dependencies and may become multimodal due to structural non-identifiability (Kappelmann *et al.* 2016, Borah Slater *et al.* 2023). Designing trans-dimensional proposals that move efficiently between model-specific flux spaces while maintaining acceptable acceptance probabilities is therefore challenging. We address this using a divide-and-conquer strategy that decomposes the full inference into tractable reaction-set-level subproblems.

### 3.1 Dividing flux inference into smaller problems

We partition the full model set ${\left\{M^{k}\right\}}_{K}$ into *K* disjoint subsets ${\left\{M^{k}\right\}}_{I^{k}}$, each corresponding to a fixed reaction set. Within each model subset, uncertainty is restricted to reaction bidirectionality, allowing the use of established RJMCMC methods (Theorell and Nöh 2020).

Expressed in terms of these subsets, the full model set-averaged flux posterior in Equation (3) is written as (see Section S.1, available as supplementary data at *Bioinformatics* online)


(6) $$\begin{array}{c} p(v_{{\left\{M^{k}\right\}}_{K}}|D)=\sum_{k^{\prime }=1}^{K}\;p({\left\{M^{k^{\prime }}\right\}}_{I^{k^{\prime }}}|D)\cdot p(v_{{\left\{M^{k^{\prime }}\right\}}_{I^{k^{\prime }}}}|D) \end{array}$$

that is, a weighted combination of model subset-averaged flux posteriors. The corresponding posterior probabilities of the model subsets are given by


(7) $$\begin{array}{c} p({\left\{M^{k}\right\}}_{I^{k}}|D)=\frac{p(D|{\left\{M^{k}\right\}}_{I^{k}})\cdot p({\left\{M^{k}\right\}}_{I^{k}})}{\sum_{k^{\prime }=1}^{K}\;p(D|{\left\{M^{k^{\prime }}\right\}}_{I^{k^{\prime }}})\cdot p({\left\{M^{k^{\prime }}\right\}}_{I^{k^{\prime }}})} \end{array}$$

The key quantity herein is the evidence of each model subset, which is obtained by aggregating the evidences of all single models within the subset


(8) $$p(D|{\left\{M^{k}\right\}}_{I^{k}})=\sum_{i^{\prime }\in I^{k}}p(M_{i^{\prime }}^{k}|{\left\{M^{k}\right\}}_{I^{k}})\cdot p(D|M_{i^{\prime }}^{k})$$

This reformulation transforms the original inference problem in Equations (3)–(5) into a finite set of *K* smaller BMA problems, each defined on a fixed reaction set and therefore amenable to RJMCMC-based inference (Theorell and Nöh 2020).

### 3.2 Conquering subset-level problems

To obtain the model set-averaged flux posterior in Equation (6), accurate estimation of the evidences $p(D|{\left\{M^{k}\right\}}_{I^{k}})$ is required. For this, we employ DNS. DNS provides a flexible framework for evidence estimation in multimodal posterior landscapes and has previously been successfully combined with RJMCMC for trans-dimensional Bayesian inference (Brewer *et al.* 2011, Brewer 2015). DNS is an iterative algorithm that uses MCMC to sample from regions of increasing likelihood. When combined with RJMCMC as the exploration mechanism, it enables sampling across both discrete model and continuous parameter spaces (Brewer *et al.* 2016). In our case, these correspond to model structure variants within a model subset and their associated flux polytopes. Embedding RJMCMC within DNS is known as trans-dimensional DNS (TDNS) (Brewer 2015, Brewer *et al.* 2016). Our key point is that restricting trans-dimensional moves to bidirectionality changes makes TDNS directly applicable to each subset-level problem. This allows us to reuse established RJMCMC proposals (Theorell and Nöh 2020) without modification and avoids the need to design new cross-model proposals across reaction sets, which would otherwise be prohibitively complex.

The resulting subset evidences are used to compute posterior model subset probabilities in Equation (7), posterior single-model probabilities (Section S.2, available as supplementary data at *Bioinformatics* online), and, by combining subset-level posteriors according to Equation (6), the full model set-averaged flux posterior. We refer to this algorithm as *Bayesian model set averaging* (BMSA), with pseudocode given in Algorithm 1. The dominant computational cost arises from the subset-level inference and evidence estimation (L 14), while the final aggregation across the *K* subsets is negligible (L 18, 21); cf. SI (Section S.2, available as supplementary data at *Bioinformatics* online) for details.

Algorithm 1 BMSA: ^13^C-MFA under model uncertainty

1: **input:** 

2:  Model set ${\left\{M^{k}\right\}}_{K}$, partitioned into *K* subsets ${\left\{M^{k}\right\}}_{I^{k}}$

3:  Flux priors $p(v_{M_{i}^{k}}|M_{i}^{k})$

4:  Model set priors $p({\left\{M^{k}\right\}}_{I^{k}})$

5:  Model priors $p(M_{i}^{k}|{\left\{M^{k}\right\}}_{I^{k}})$

6:  Data D

7: **output:** 

8:  Full model set-averaged flux posterior $p(v_{{\left\{M^{k}\right\}}_{K}}|D)$

9:  Model subset probabilities $p({\left\{M^{k}\right\}}_{I^{k}}|D)$

10:   Single-model probabilities (subset) $p(M_{i}^{k}|{\left\{M^{k}\right\}}_{I^{k}},D)$

11:   Single-model probabilities (full set) $p(M_{i}^{k}|{\left\{M^{k}\right\}}_{K},D)$

12: **procedure** BMSA:

13:  **for**  $k^{\prime }\leftarrow 1$ to *K* **do** 

14:   $\begin{array}{cc} p(v_{{\left\{M^{k^{\prime }}\right\}}_{I^{k^{\prime }}}}|D) & \;\;▹\;\text{Eq}.\;(1)\\ p(D|{\left\{M^{k^{\prime }}\right\}}_{I^{k^{\prime }}}) & \;\;▹\;\text{Eq}.\;(8) \end{array}\}\leftarrow \text{TDNS},\text{SI}\;S.2$

15:   **for all**  $i^{\prime }\in I^{k^{\prime }}$  **do** 

16:    $p(M_{i^{\prime }}^{k^{\prime }}|{\left\{M^{k^{\prime }}\right\}}_{I^{k^{\prime }}},D)\leftarrow \frac{\#\text{samples}(M_{i^{\prime }}^{k^{\prime }})}{\#\text{samples}({\left\{M^{k^{\prime }}\right\}}_{I^{k^{\prime }}})}$

17:   **end for** 

18:   **return**  $p({\left\{M^{k^{\prime }}\right\}}_{I^{k^{\prime }}}|D)$  ▹ Eq. (7)

19:   **return**  $\begin{array}{c} p(M_{i^{\prime }}^{k^{\prime }}|{\left\{M^{k}\right\}}_{K},D)\leftarrow \\ p(M_{i^{\prime }}^{k^{\prime }}|{\left\{M^{k^{\prime }}\right\}}_{I^{k^{\prime }}},D)\cdot p({\left\{M^{k^{\prime }}\right\}}_{I^{k^{\prime }}}|D) \end{array}$

20:  **end for** 

21:  **return**  $p(v_{{\left\{M^{k}\right\}}_{K}}|D)$  ▹ Eq. (6)

22:  **end procedure** 

BMSA has the advantageous property that the loop over $k^{\prime }$ (L 13) is embarrassingly parallel, enabling independent subset-level inference across multi-core and multi-node HPC architectures. The partition can also be refined to match available compute resources, provided models from different reaction sets remain in separate subsets. Together, these properties make BMSA scalable across large model families.

### 3.3 Implementation

We implemented BMSA for ^13^C-MFA using four open-source packages. (i) For DNS we use the high-performance implementation DNest4 (Brewer and Foreman-Mackey 2018). DNest4 (v0.2.4) allows us to implement custom likelihood functions and proposal mechanisms. We developed a fork that improves performance for ^13^C-MFA model sampling, provides robust checkpointing on HPC systems, and stores samples exactly using hexadecimal floating-point output (https://github.com/modsim/dnest4). (ii) For convex polytope sampling we use hopsy (v1.7.0) (Paul *et al.* 2024), which builds on the C++ library HOPS for optimized sampling in high-dimensional polytopes (Jadebeck *et al.* 2021). A custom adapter connects hopsy to DNest4, allowing direct reuse of our RJMCMC proposals. (iii) ^13^C labeling data are simulated using the high-performance simulator 13CFLUX (v3.0.0a) (Stratmann *et al.* 2025). Its high simulation performance is essential because likelihood evaluations dominate the cost of Bayesian sampling procedures for realistic ^13^C-MFA models. Although each of the above libraries provides a Python interface, we use their native C++ APIs to minimize overhead. (iv) Models are preprocessed with PolyRound (v0.3.0) (Theorell *et al.* 2022), which removes redundant constraints and computes a maximum volume ellipsoid to round the feasible flux polytopes (Jadebeck *et al.* 2023).

## 4 Application studies

To evaluate the BMSA algorithm, in this work we address three questions:

1. Does the method yield correct and interpretable results?
2. Is our implementation computationally feasible for real-world problems?
3. How does data informativeness influence model uncertainty and flux inferences?

We investigate these aspects with two case studies: a minimal toy system for intuitive validation under controlled conditions, and an application-scale *Escherichia coli* model (Long and Antoniewicz 2019) for assessing scalability and inference under realistic levels of data informativeness, controlled through tracer selection. Because the true metabolic state is unknown for real data, we use synthetic datasets generated from published models by simulating measurements for the same metabolites as in the published datasets to quantify model and flux recovery against known ground truth.

### 4.1 Illustrative example: *Triangulus*

**Figure 1:**
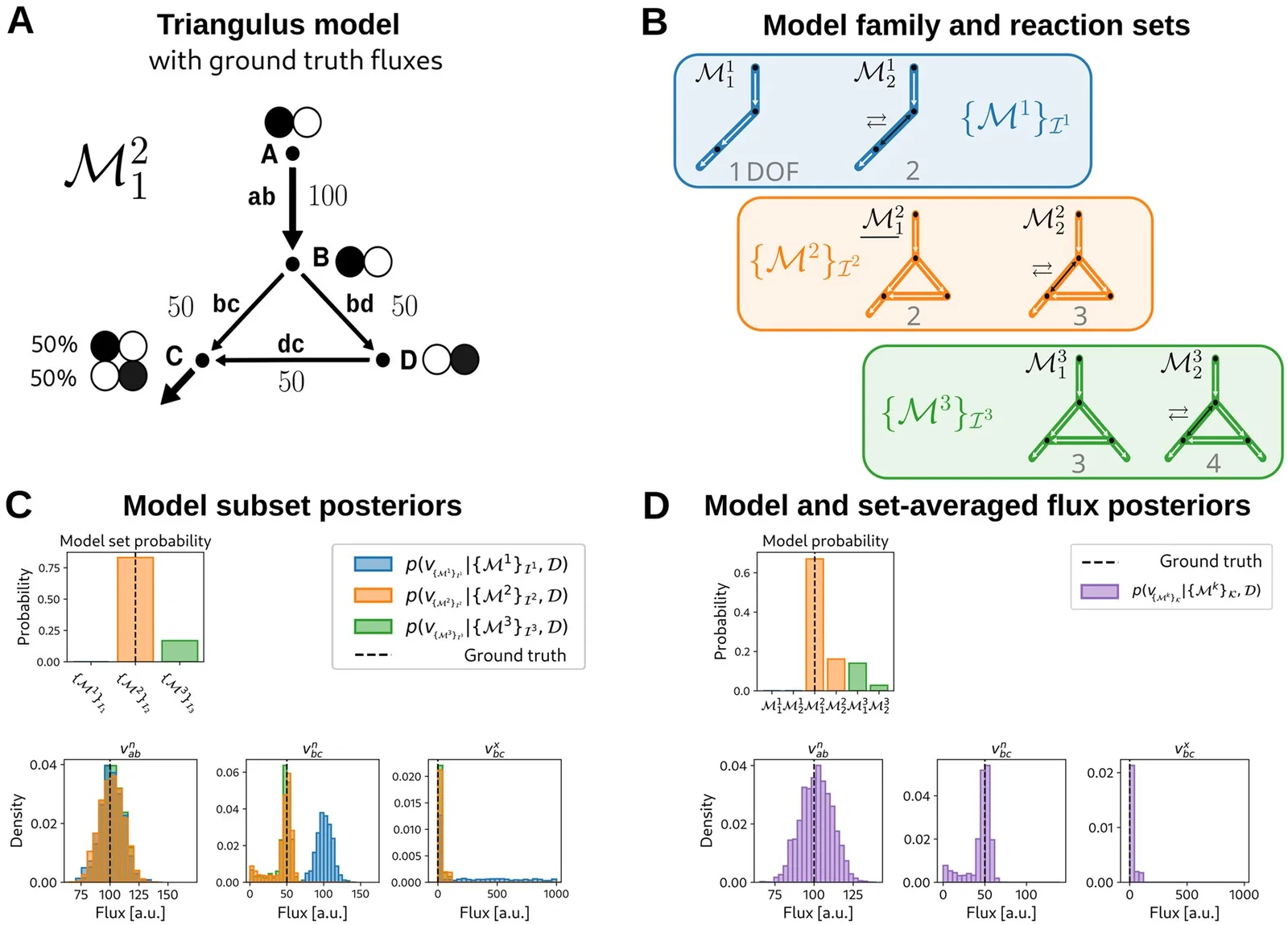
*Triangulus* case study. (A) Ground truth network with ILE data for the positionally labeled tracer (A●∘). Due to the scrambling reaction bd, the labeling pattern of C reflects the flux ratio between the two pathways. (B) Model variants and their reaction sets; within each subset models differ by uni- or bidirectionality of reaction bc. (C) Inference results at the model subset level. Subset posterior probabilities and corresponding marginal flux posteriors are color-coded. The blue subset lacks reactions bd and dc, constraining $v_{bc}^{n}$ to equal $v_{ab}^{n}$. (D) Inference results on the full model set level, showing posterior probabilities of individual models and model-averaged marginal flux posteriors. See Fig. S4, available as supplementary data at *Bioinformatics* online for convergence diagnostics.

We first apply BMSA to a minimal network, referred to as *Triangulus*, capturing key features of ^13^C-MFA models: a parallel pathway between a source metabolite (A) and an observable metabolite (C) (cf. Fig. 1A). The connection proceeds either via an unidirectional two-step route via metabolite B (ab-bc) or an unidirectional three-step route involving metabolites B and D (ab-bd-dc). While the direct route preserves atom positions, the alternative pathway introduces scrambling, making the labeling of C informative about the flux ratio between the net fluxes $v_{bc}^{n}$ and $v_{bd}^{n}$ (with $v_{bd}^{n}$=$v_{dc}^{n}$) when using a positionally labeled substrate.

Synthetic data were generated using the positionally labeled tracer A•∘ and a reference flux configuration with equal flux through both pathways. Mass isotopologues of the first and second C fragments were simulated, and domain-typical measurement noise was added to the uptake flux $v_{ab}^{n}$ (10% relative Gaussian error) and the labeling data (standard deviation of 0.01). In this setup, the data provide sufficient information to identify the two independent net fluxes under the assumed models.

To introduce structural uncertainty, we construct a small model family (cf. Fig. 1B) by allowing reaction bc to be bidirectional and by introducing an additional export reaction for D. The resulting model set comprises under-complex variants ($M_{1}^{1}$ DOF = 1), the ground-truth *Triangulus* model $M_{1}^{2}$ (DOF = 2), a second model with two independent flux parameters ($M_{2}^{1}$), and three more flexible over-parametrized model variants ($M_{2}^{2}$, $M_{1}^{3}$, $M_{2}^{3}$ with DOF = 3–4). By construction, these models form three reaction sets (blue, orange, green), which also define the BMSA model set partitioning. Model and flux priors for all models, as well as conventional single-model flux posteriors for reference are provided in the SI (cf. Table S1, Figs. S2 and S3, available as supplementary data at *Bioinformatics* online).

Applying BMSA, we first analyze the inferred model subset probabilities and subset-averaged flux posteriors (Fig. 1C). As expected, the blue subset receives effectively zero probability, confirming that its models cannot explain the data. The orange and green subsets both provide viable explanations for the data, with the orange subset (containing the ground-truth model) dominating with ∼83%, compared to ∼17% for the green subset. This reflects an Ockham’s razor (MacKay, 2008; McFadden, 2023): although both subsets can explain the data equally, only a small fraction of the larger flux space of the more flexible models of the green subset yields flux solutions consistent with the measurements, reducing the evidence of the green subset.

Subset-averaged flux posteriors further illustrate this. The uptake flux $v_{ab}^{n}$, which is directly measured, is accurately recovered by all models, including its uncertainty. In contrast, models in the blue subset produce biased estimates for internal fluxes due to their inability to reproduce the data. The bidirectionality of reaction bc in $M_{2}^{1}$ remains undecidable, with approximately equal posterior support for being uni- and bidirectional, reflected in the distribution of exchange flux values $v_{bc}^{x}$. Both the orange and green subsets explain the labeling data and therefore yield nearly identical subset-averaged flux posteriors, with most probability mass concentrated around the ground truth values. The lower tails of the orange and green $v_{bc}^{n}$ posteriors arise from non-zero exchange flux values $v_{bc}^{x}$, which introduce label cycling as an alternative, though less probable, explanation for the C labeling measurements.

Combining these subset-related results yields model probabilities in view of the full model set and the full model set-averaged flux posterior (cf. Fig. 1D). The under-complex blue models receive negligible probability, while the model evidence balances fit quality against flexibility among the remaining models. Consequently, the ground truth model $M_{1}^{2}$ dominates (∼66% probability), followed by more flexible ones, the orange model $M_{2}^{2}$ (∼16%) and the two green models $M_{1}^{3}$ (∼14%) and $M_{2}^{3}$ (∼3%).

Overall, the posterior favors the simplest model that adequately explains the data (the ground truth model $M_{1}^{2}$). As a result, the full model-averaged flux posterior closely matches the generating flux configuration. Thus, under sufficiently informative data, BMSA yields interpretable results, balances fit against flexibility, and recovers the ground truth.

### 4.2 Realistic example: *Escherichia coli*

Although the *Triangulus* example provides intuition, practical ^13^C-MFA models comprise dozens to hundreds of reactions, combinatorial model families that may reach millions or more variants, and experimental data that rarely resolve every flux or model structure. We therefore apply BMSA to the published *Escherichia coli* Δ  *tpiA* study of Long and Antoniewicz (2019). In this mutant, residual TPI activity after deletion of the triosephosphate isomerase gene is nearly zero, the methylglyoxal (MGOX) pathway prevents dihydroxyacetone phosphate accumulation, the glyoxylate shunt (GOX) is active, and the Entner-Doudoroff pathway (EDP) carries only a small net flux. This system therefore constitutes a challenging test case involving several latent pathways in central metabolism.

We reconstructed the published network and fixed both $v_{\mathrm{TPI}}^{n}$ and $v_{\mathrm{TPI}}^{x}$ (denoted TPI pathway) to zero (Fig. S5, available as supplementary data at *Bioinformatics* online). The reference model contains 81 metabolites and 126 reactions, with 11 independent net fluxes and 23 independent exchange fluxes (DOF = 34). We introduced structural model uncertainty by treating TPI, EDP, GOX, and MGOX pathway activity as uncertain. This yields 12 reaction sets: eight when TPI is active, while MGOX, EDP and GOX may be active or inactive, and four when TPI is inactive, in which case MGOX must remain active (cf. Table S2, available as supplementary data at *Bioinformatics* online). Combined with uncertain bidirectionality, the candidate family comprises 46 976 204 800 models spanning 9–46 DOF. This defines an application-scale benchmark with ground truth and realistic pathway-level uncertainty. Model subsets correspond to reaction sets (cf. Table S2, available as supplementary data at *Bioinformatics* online) and priors are specified in Section S.4.3, available as supplementary data at *Bioinformatics* online.

#### 4.2.1 Single-ILE evaluation

**Figure 2:**
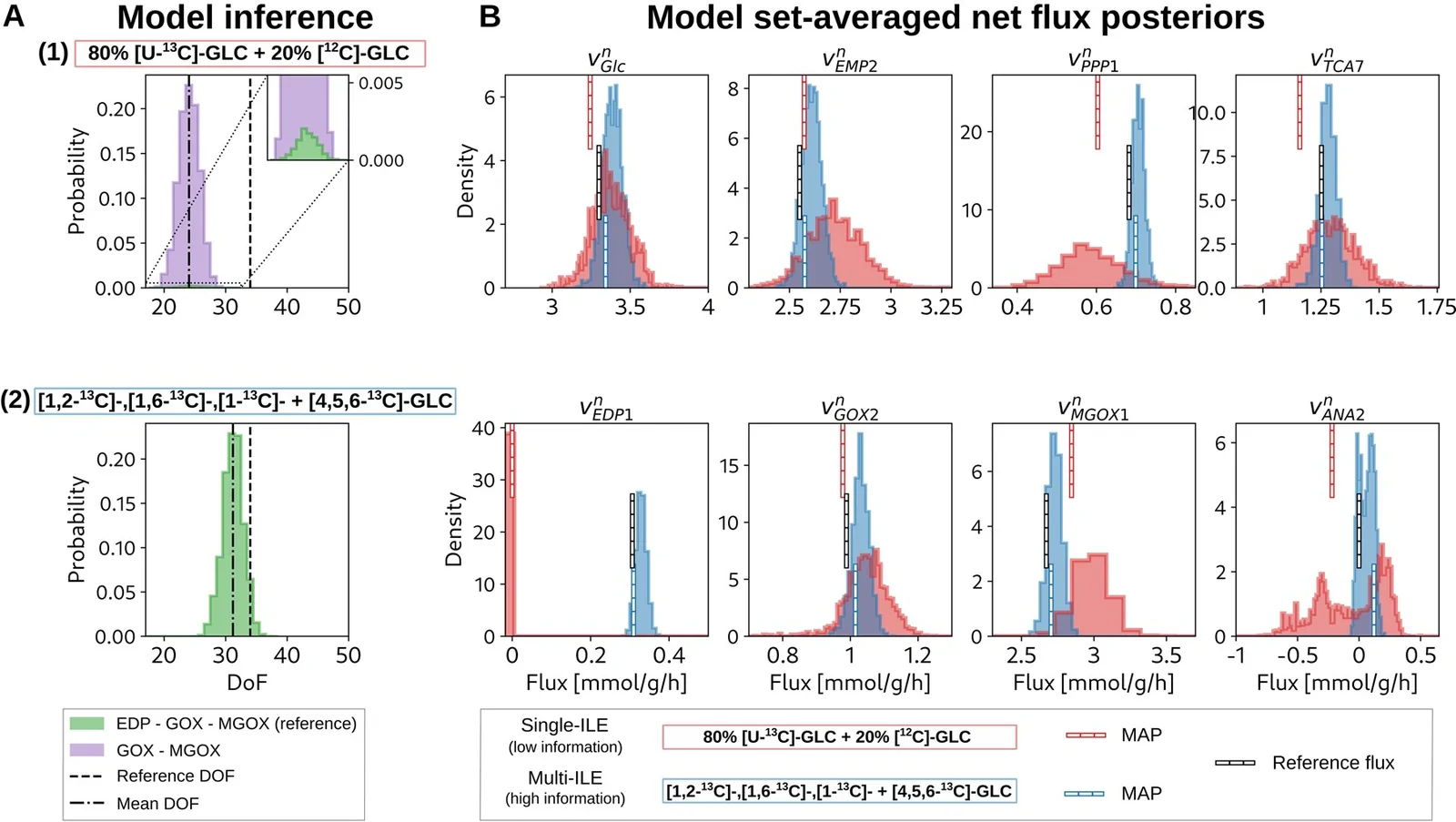
Inference results for the *Escherichia coli* case study. (A) Stacked histogram of posterior model probability across effective model DOF. Each bar represents the total posterior probability of all model variants with a given number of DOF, partitioned by model subset (color-coded). In the single-ILE evaluation, the subset containing the reference model receives only ∼0.9%, whereas the simpler model subset with active GOX and MGOX and inactive TPI and EDP dominates (99.1%). The DOF of the reference model is indicated by the dashed line. In the multi-ILE analysis (lower panel), the reference reaction set is recovered with >99.9% probability. (B) Model set-averaged marginal posteriors for selected net fluxes, including maximum a posteriori (MAP) estimates. Results for all 75 net fluxes are shown in Fig. S6, available as supplementary data at *Bioinformatics* online. Inferences are reproducible across independent BMSA runs (cf. Section S.4.6, available as supplementary data at *Bioinformatics* online).

We first consider a typical experimental scenario using the relatively uninformative tracer mixture 80% [U-^13^C]+20% [^12^C] glucose. Synthetic data were generated from the reference model and perturbed using the noise levels reported in the original study. Fig. 2A(1) summarizes the model inference in terms of DOF distribution over the models that carry the majority of posterior probability mass. We define the *effective model set* as the smallest collection of models with cumulative posterior probability of at least 95%, thereby effectively contributing to the model set-averaged flux posterior. Despite the 46 976 204 800 candidates, this effective model set contains only 1572 models ($3.3\times 10^{-6}$%), indicating strong posterior concentration. All effective models share the reaction set with active GOX and MGOX pathways (plum), which receives 99.1% posterior probability, whereas the reference reaction set with active EDP, MGOX, and GOX receives only ∼0.9%. Thus, the applied tracer does not provide sufficient support for EDP activity.

This outcome reflects limited informativeness of the data. Because the model evidence averages the likelihood over the flux polytope, additional reactions or pathways are favored only when the improvement in explaining the data compensates for the increased flux space (Ockham’s razor). Here, the labeling data do not sufficiently support the additional DOF introduced by the EDP, down-weighting models in which the EDP is active. Importantly, this reflects a lack of statistical support under the given data and priors, rather than evidence of biological inactivity.

Consistently, the effective model set has substantially fewer DOF than the reference model (24.1±1.6 vs. 34). EDP deactivation accounts for only one of the reduced DOF; the remaining ∼ 9 DOF are attributed to unresolved bidirectionalities. On average, only 14.1±1.6 of the 23 bidirectional reactions in the reference model are inferred as bidirectional. This quantifies the limited information content in the data and illustrates how BMA deals with structural model uncertainty rather than ignoring it.

The corresponding model set-averaged marginal flux posteriors (Fig. 2B) are mostly unimodal (cf. Fig. S6, available as supplementary data at *Bioinformatics* online for the remaining net fluxes). The exception is malate dehydrogenase (ANA2), whose bimodal posterior indicates alternative anaplerotic modes, previously reported theoretically (Kappelmann *et al.* 2016) and experimentally (Borah Slater *et al.* 2023). Because models without EDP dominate the effective model set, fluxes in EMP and PPP shift relative to the reference values. Nevertheless, uncertainty bounds generally encompass the reference fluxes. Interestingly, for several EMP and PPP fluxes, uncertainty is even lower than under single-model inference (cf. Fig. S7, available as supplementary data at *Bioinformatics* online), because the data favor models with fewer DOF and hence effectively restrict the admissible flux spaces.

The BMSA run converged after 147 456 core-hours (4 days) on JURECA (cf. Fig. S9, available as supplementary data at *Bioinformatics* online), demonstrating feasibility for an application-scale problem. The reported runtime reflects the cost of statistically rigorous Bayesian evidence estimation across a large structural model space. Its computational cost depends primarily on the number of candidate models and the complexity of the posterior, rather than directly on the size of the reaction network. Notably, despite explicitly accounting for structural uncertainty, the resulting flux uncertainties do not inflate even under the weakly informative ILE dataset. Instead, BMSA down-weights unsupported model flexibility, so that only structural uncertainty supported by the data is propagated into the flux estimates.

#### 4.2.2 Multi-ILE evaluation

We next assess how increased data informativeness changes the inferences by jointly analyzing three ILE datasets with complementary tracers (100% [1,2-^13^C], 100% [1,6-^13^C], and 50% [1-^13^C] + 50% [4,5,6-^13^C]). These tracers resolve different parts of central metabolism, tripling the number of measurements without changing the models’ DOF. We therefore expect improved recovery of the reference model structure and more precise flux estimates.

The joint analysis markedly changes the posterior landscape (cf. Fig. 2A(2) and B). The effective model set contains 1205 models ($2.6\times 10^{-6}\%$ of all candidates), ∼23% fewer than in the single-ILE case. The reference reaction set (EDP-GOX-MGOX) now receives >99.9% posterior probability. The DOF distribution shifts upward and centers at 31.2±1.7, approaching the reference DOF number (34). Thus, the additional data support the previously unresolved EDP activity and reduce bidirectionality uncertainty. Interestingly, we found a slightly increased DOF spread compared to the single-ILE case, which reflects residual uncertainty in the two unresolved reaction bidirectionalities.

The flux posteriors are correspondingly sharper and closely match the reference flux map (cf. Fig. S6, available as supplementary data at *Bioinformatics* online). In particular, the previously bimodal ANA2 net flux posterior collapses to a single mode, resolving the alternative anaplerotic explanations. This sharpening occurs despite explicitly accounting for model uncertainty. The multi-ILE analysis was obtained using 405 504 core-hours (11 days) on JURECA (cf. Fig. S9, available as supplementary data at *Bioinformatics* online), demonstrating feasibility for integrated multi-dataset inference.

Overall these experiments show that BMSA adapts the effective model set complexity to the information content of the data: unsupported flexibility is penalized, whereas informative data support pathway recovery and precise, but not overconfident, flux estimates (cf. Fig. S8, available as supplementary data at *Bioinformatics* online for a comparison with conventional single-model inference). Posterior model probabilities therefore provide a principled measure of relative support within the specified model family, enabling evidence-based identification of pathways statistically supported by the data.

## 5 Conclusion

We presented BMSA, a scalable BMA algorithm for ^13^C-MFA that quantifies metabolic fluxes while explicitly accounting for pathway- and reaction-level model uncertainty through evidence-based model-set weighting. Rather than relying on a single selected model, BMSA integrates over a spectrum of plausible models, yielding interpretable posterior support for pathway hypotheses and flux posteriors that reflect both parameter and structural uncertainty.

Rather than seeking a single “correct” model, it quantifies which structural hypotheses are supported by the available data and propagates the resulting uncertainty into the flux estimates. Algorithmically, BMSA combines RJMCMC for trans-dimensional model exploration with DNS for robust evidence estimation, enabling principled inferences across large, nonlinear, and polytope-constrained model and parameter settings. This is particularly relevant for ^13^C-MFA, where uncertain pathways and reaction bidirectionalities can otherwise lead to model-dependent conclusions. Consistent with evidence-based inference, pathway hypotheses with weak data support are down-weighted despite remaining biologically active, reflecting limited identifiability rather than biological inactivity. Conversely, increasing data informativeness recovers the correct model configuration and sharpens flux estimates without committing to unsupportable assumptions.

We demonstrated correctness and interpretability on a toy example and computational feasibility on an application-scale *E. coli* benchmark spanning billions of model variants. Owing to its divide-and-conquer formulation, BMSA is naturally parallelizable, and its computational performance can be improved further through model-set partitioning and algorithmic optimization. While computationally demanding, it yields statistically rigorous information on structural uncertainty that is inaccessible to conventional single-model analyses, an investment that is particularly justified in ^13^C-MFA, where experimental data acquisition is costly and limited.

Together, these results establish BMSA as a practical and extensible framework for Bayesian flux inference under structural model uncertainty. By replacing fixed structural modeling assumptions with statistically testable hypotheses, BMSA enhances the transparency, robustness, and interpretability of ^13^C-MFA analyses and provides a principled basis for uncertainty-aware quantitative metabolic studies.

## Supplementary Material

btag587_Supplementary_Data
