# Supplementary material for: Trans-dimensional Bayesian model averaging for 13C-metabolic flux analysis: evidence-based flux inference under structural model uncertainty
Source: Bioinformatics. 2026 Aug 4;42(9):btag587. doi: 10.1093/bioinformatics/btag587 (PMC13549947; doi:10.1093/bioinformatics/btag587)
Supplement: Supplementary file 1 [file btag587_supplementary_data.pdf]

Supplementary Information for

Trans-dimensional Bayesian model averaging for ^{13}C-metabolic flux analysis: Evidence-based flux inference under structural model uncertainty

Johann F. Jadebeck [1,2], Anton Stratmann [1,2], Martin Beyß [1,2], and Katharina Nöh [1]

[1]Institute of Bio- and Geosciences, IBG-1: Biotechnology, Forschungszentrum Jülich, 52428 Jülich, Germany

[2]Computational Systems Biotechnology (AVT.CSB), RWTH Aachen University, 52074 Aachen, Germany

Email: k.noeh@fz-juelich.de

Contents

S.1 BMA formulation for model sets **2**

S.2 Trans-dimensional diffusive nested sampling **3**
S.2.1 The augmented flux space for reversible-jump MCMC 3
S.2.2 Model set evidence 3
S.2.3 Nested sampling background 4
S.2.4 TDNS for BMSA 6

S.3 *Triangulus*: Additional information **11**
S.3.1 Prior specification 11
S.3.2 Marginal posteriors 13
S.3.3 TDNS diagnostics 14

S.4 *Escherichia coli* use case: Additional information **15**
S.4.1 Reference model and flux map 15
S.4.2 Model subsets 16
S.4.3 Prior specification 17
S.4.4 Marginal posteriors 18
S.4.5 TDNS for BMSA parameters & diagnostics 21
S.4.6 Reproducibility of results 23

Nomenclature **25**

Scripts and model files are available at https://doi.org/10.5281/zenodo.21324804.

S.1 BMA formulation for model sets

The BMA formulation to determine the model set-averaged flux posterior $v_{\{\mathcal{M}^k\}_\mathcal{K}}$ in view of the full model set $\{\mathcal{M}^k\}_\mathcal{K}$

$$p(v_{\{\mathcal{M}^k\}_\mathcal{K}} \mid \mathcal{D}) = \sum_{k'=1}^{K} p(\{\mathcal{M}^{k'}\}_{\mathcal{I}^{k'}} \mid \mathcal{D}) \cdot p(v_{\{\mathcal{M}^{k'}\}_{\mathcal{I}^{k'}}} \mid \mathcal{D}) \tag{S.1}$$

is equivalent to

$$p(v_{\{\mathcal{M}^k\}_\mathcal{K}} \mid \mathcal{D}) = \sum_{\substack{k'=1,\\ i'\in\mathcal{I}^{k'}}}^{K} p(\mathcal{M}_{i'}^{k'} \mid \mathcal{D}) \cdot p(v_{\mathcal{M}_{i'}^{k'}} \mid \mathcal{D}) \tag{S.2}$$

where in contrast to the main text, we have suppressed the conditioning of $p(\mathcal{M}_{i'}^{k'}|\mathcal{D})$ on the (fixed) model set $\{\mathcal{M}^k\}_\mathcal{K}$.
To prove the equivalence of Eq. (S.1) and Eq. (S.2), we condition the probability of all models $\mathcal{M}_i^k$ on their containing model subset $\{\mathcal{M}^k\}_{\mathcal{I}^k}$

$$p(\mathcal{M}_i^k \mid \mathcal{D}) = p(\{\mathcal{M}^k\}_{\mathcal{I}^k} \mid \mathcal{D}) \cdot p(\mathcal{M}_i^k \mid \{\mathcal{M}^k\}_{I^k}, \mathcal{D}),\ \forall i \in \mathcal{I}^k, k \in \{1, \ldots, K\} \tag{S.3}$$

It then follows

$$\begin{aligned}
p(v_{\{\mathcal{M}^k\}_\mathcal{K}} \mid \mathcal{D}) &\overset{Eq.\ (S.2)}{=} \sum_{\substack{k'=1\\ i'\in\mathcal{I}k'}}^{K} p(\mathcal{M}_{i'}^{k'} \mid \mathcal{D}) \cdot p(v_{\mathcal{M}_{i'}^{k'}} \mid \mathcal{D}) \\
&\overset{Eq.\ (S.3)}{=} \sum_{\substack{k'=1\\ i'\in\mathcal{I}^{k'}}}^{K} p(\{\mathcal{M}^{k'}\}_{\mathcal{I}^{k'}} \mid \mathcal{D}) \cdot p(\mathcal{M}_{i'}^{k'} \mid \{\mathcal{M}^{k'}\}_{\mathcal{I}^{k'}}, \mathcal{D}) \cdot p(v_{\mathcal{M}_{i'}^{k'}} \mid \mathcal{D}) \\
&= \sum_{k'=1}^{K} p(\{\mathcal{M}^{k'}\}_{\mathcal{I}^{k'}} \mid \mathcal{D}) \cdot \sum_{i'\in\mathcal{I}^{k'}} p(\mathcal{M}_{i'}^{k'} \mid \{\mathcal{M}^{k'}\}_{\mathcal{I}^{k'}}, \mathcal{D}) \cdot p(v_{\mathcal{M}_{i'}^{k'}} \mid \mathcal{D}) \\
&\overset{Eq.\ (1)}{=} \sum_{k'=1}^{K} p(\{\mathcal{M}^{k'}\}_{\mathcal{I}^{k'}} \mid \mathcal{D}) \cdot p(v_{\{\mathcal{M}^{k'}\}_{\mathcal{I}^{k'}}} \mid \mathcal{D})
\end{aligned}$$

where we use the BMA formulation for the model set $\{\mathcal{M}\}_{\mathcal{I}^k}$ in Eq. (1) of the main manuscript, yielding the equivalence of Eq. (S.1) and Eq. (S.2).

S.2 Trans-dimensional diffusive nested sampling

The computational core of Bayesian model set averaging (BMSA) given in Algorithm 1 in the main text builds on trans-dimensional diffusive nested sampling (TDNS). We adapt the TDNS algorithm in [4] to the mixed discrete-continuous flux inference problem over model subsets $\{\mathcal{M}^k\}_{\mathcal{I}^k}$. While Algorithm 1 in the main text describes how the results for the model subsets are combined to the model set-averaged flux posterior over the full model set, we here concentrate on the results per model subset. For brevity, we speak of the model set $\{\mathcal{M}^k\}_{\mathcal{I}^k}$ instead of model subset in the following.
Given observed data $\mathcal{D}$ and the model set $\{\mathcal{M}^k\}_{\mathcal{I}^k}$, TDNS for BMSA provides

- *model set-averaged flux posterior* $p(v_{\{\mathcal{M}^k\}_{\mathcal{I}^k}} \mid \mathcal{D})$
- *model set evidences* $p(\mathcal{D} \mid \{\mathcal{M}^k\}_{\mathcal{I}^k})$

These quantities enable the computation of posterior model set probabilities and the full Bayesian model-averaged flux posteriors as described in the main text. As by-product, samples approximating the single-model posterior $p(\mathcal{M}_i^k \mid \{\mathcal{M}^k\}_{\mathcal{I}^k}, \mathcal{D})$ in view of the considered model set $\{\mathcal{M}^k\}_{\mathcal{I}^k}$ are generated.

The TDNS for BMSA algorithm combines ideas from diffusive nested sampling (DNS) and reversible-jump Markov chain Monte Carlo (RJMCMC), exploiting the specific structure of the flux inference problem. In next sections, we summarize the essential underlying concepts: (i) the state space for RJMCMC, which we call the augmented flux space (Section S.2.1), and (ii) the model set evidence integral over the augmented flux space (Section S.2.2). In Section S.2.3, we introduce necessary background to nested sampling, in particular, the integral transform required to approximate the integral of the model set evidence. Finally, we describe TDNS for BMSA in in Section S.2.4, along with pseudocode.

S.2.1 The augmented flux space for reversible-jump MCMC

The model set $\{\mathcal{M}^k\}_{\mathcal{I}^k}$ consists of models $\mathcal{M}_i^k$ with the same reactions, but varying reaction bidirectionality [13]. Therefore, each model is equipped with a model-specific set of (independent) fluxes $v_{\mathcal{M}_i^k}$ that are defined on the model-specific flux polytope $\mathcal{P}_{\mathcal{M}_i^k}$. The dimension of the flux polytope, which corresponds to the DOF, may vary between different models, depending on their bidirectionality setting. By zeroing exchange fluxes for unidirectional reactions, the fluxes $v_{\mathcal{M}_i^k}$ can be embedded in a common ambient flux representation, while the DOF remains specific to the model.
TDNS uses RJMCMC [13] to traverse the discrete-continuous space of models and model-specific fluxes. Before we describe TDNS, we formally introduce the discrete-continuous space of models and model-specific fluxes. We define the *augmented flux space* $\Omega_{\{\mathcal{M}^k\}_{\mathcal{I}^k}}$ as disjoint union over the Cartesian products of all single models and their associated flux polytopes

$$\Omega_{\{\mathcal{M}^k\}_{\mathcal{I}^k}} = \bigcup_{i \in \mathcal{I}^k} \{i\} \times \mathcal{P}_{\mathcal{M}_i^k} \tag{S.4}$$

An element of the augmented flux space is thus represented as a tuple $(i, v_{\mathcal{M}_i^k})$ with $i \in \mathcal{I}^k$ the model index and fluxes $v_{\mathcal{M}_i^k}$ in the associated flux polytope.

S.2.2 Model set evidence

The evidence of the model set $\{\mathcal{M}^k\}_{\mathcal{I}^k}$ is given by the integral of the model set specific likelihood $\mathcal{L}_{\{\mathcal{M}^k\}_{\mathcal{I}^k}}$ with respect to the prior measure $\mu_{\{\mathcal{M}^k\}_{\mathcal{I}^k}}$ related to the model set. For this, we formally extend the single model likelihoods defined on the model-specific polytopes, to the model set related likelihood $\mathcal{L}_{\{\mathcal{M}^k\}_{\mathcal{I}^k}}$ on the augmented flux space $\Omega_{\{\mathcal{M}^k\}_{\mathcal{I}^k}}$ in the sense that for each element of $\Omega_{\{\mathcal{M}^k\}_{\mathcal{I}^k}}$ there is a well-defined single model likelihood.

The model set-related prior measure $\mu_{\{\mathcal{M}^k\}_{\mathcal{I}^k}}$ is then defined by accumulating the priors of the single models that constitute the model set, weighted by the models' priors given the model set

$$\mu_{\{\mathcal{M}^k\}_{\mathcal{I}^k}} : \Omega_{\{\mathcal{M}^k\}_{\mathcal{I}^k}} \to [0,1] \text{ with}$$
$$\mu_{\{\mathcal{M}^k\}_{\mathcal{I}^k}}(A) = \sum_{i\in\mathcal{I}^k} p(\mathcal{M}_i^k \mid \{\mathcal{M}^k\}_{\mathcal{I}^k}) \cdot \int_{\mathcal{P}_{\mathcal{M}_i^k}} \mathbb{I}_A(i, v_{\mathcal{M}_i^k}) \cdot p(v_{\mathcal{M}_i^k} \mid \mathcal{M}_i^k)\, dv_{\mathcal{M}_i^k} \tag{S.5}$$

with A a measurable subset of $\Omega_{\{\mathcal{M}^k\}_{\mathcal{I}^k}}$ and the indicator function $\mathbb{I}_A$ being 1 for all tuples $(i, v_{\mathcal{M}_i^k}) \in A$ and 0 else.
Given the prior measure, the evidence of a model set $\{\mathcal{M}^k\}_{\mathcal{I}^k}$ is given by

$$p(\mathcal{D}|\{\mathcal{M}^k\}_{\mathcal{I}^k}) = \int_{\Omega_{\{\mathcal{M}^k\}_{\mathcal{I}^k}}} \mathcal{L}_{\{\mathcal{M}^k\}_{\mathcal{I}^k}}(i, v_{\mathcal{M}_i^k})\, d\mu_{\{\mathcal{M}^k\}_{\mathcal{I}^k}}(i, v_{\mathcal{M}_i^k}) \tag{S.6}$$

where $\mathcal{L}_{\{\mathcal{M}^k\}_{\mathcal{I}^k}}(i, v_{\mathcal{M}_i^k})$ is the likelihood of model $\mathcal{M}_i^k$ evaluated for fluxes $v_{\mathcal{M}_i^k}$.

S.2.3 Nested sampling background

Determining the model set evidence in Eq. (S.6) is computationally challenging. To make the calculation tractable, the key idea of nested sampling is to transform the high-dimensional evidence integral into a one-dimensional integral [12]. Figure S.1 provides a conceptual visualization of the underlying transformation.

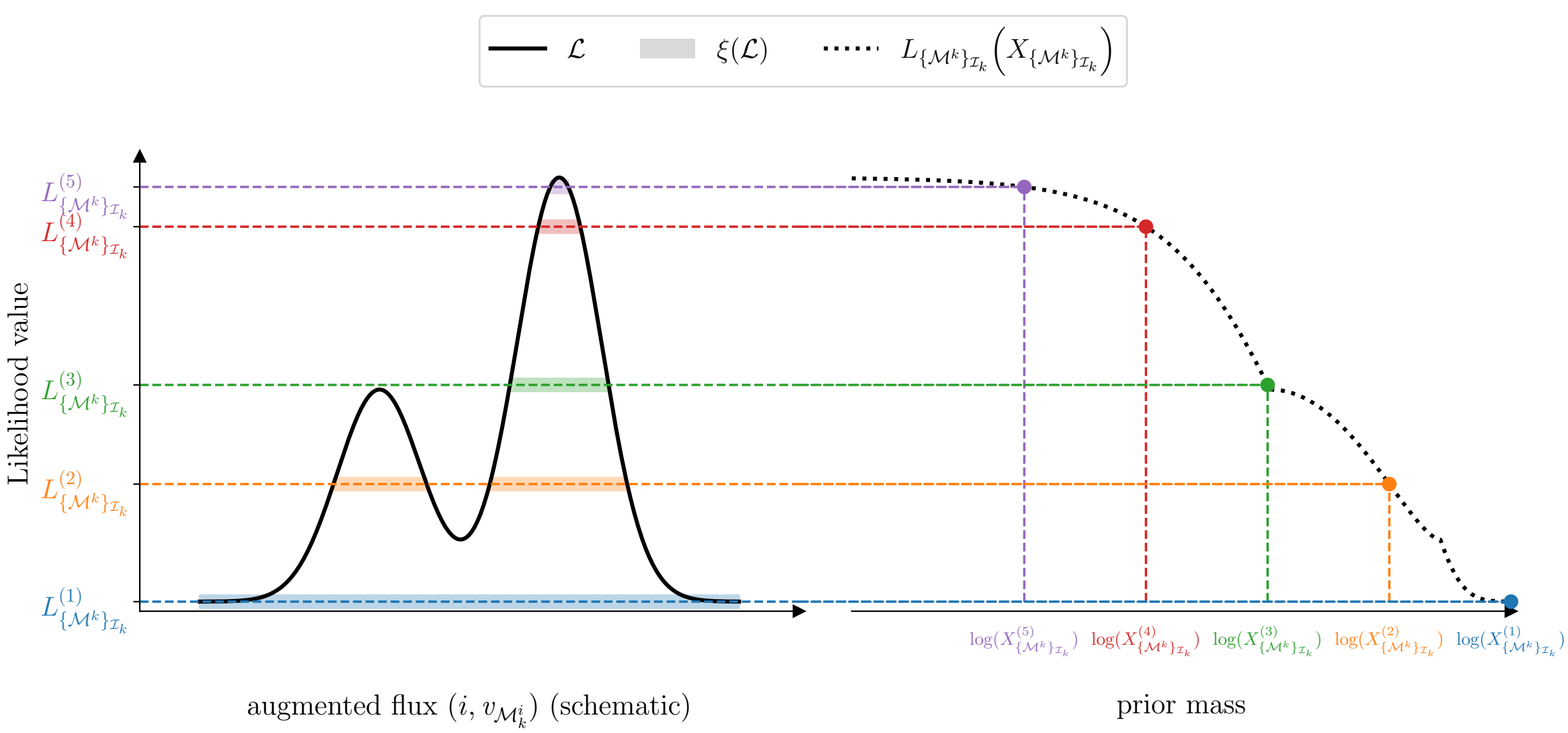

Figure S.1: Schematic representation of the evidence integral transform that nested sampling exploits. Left: Likelihood (black solid) shown as a function of model-specific fluxes. The fluxes are here formally represented as a tuple, being an element of the augmented flux space. Colored domains represent the surviving prior mass ξ for different likelihood values $L_{\{\mathcal{M}^k\}_{\mathcal{I}^k}}$. The length of the colored domains corresponds to their prior measure $X_{\{\mathcal{M}^k\}_{\mathcal{I}^k}}$. In nested sampling, it is common practice to show the likelihood in log space. For this representation, we have chosen to display the likelihood value rather than the log likelihood.
Right: Likelihood (black dotted) as a function of the prior mass. The likelihood levels on the left correspond to different values of the prior-mass coordinate $X_{\{\mathcal{M}^k\}_{\mathcal{I}^k}}$ (colored dots, prior-mass coordinates are depicted in log space). Kinks in the transformed likelihood emerge at likelihood values, where the likelihood goes from unimodal to bimodal and vice versa. These kinks are known as "phase-transitions" and occur when the surviving prior mass ξ changes sharply at a certain likelihood level.

Following the standard nested sampling formulation in [1], we rewrite the model set evidence in Eq. (S.6) as

$$p(\mathcal{D}|\{\mathcal{M}^k\}_{\mathcal{I}^k}) = \int_0^1 L_{\{\mathcal{M}^k\}_{\mathcal{I}^k}}(X_{\{\mathcal{M}^k\}_{\mathcal{I}^k}})\, dX_{\{\mathcal{M}^k\}_{\mathcal{I}^k}} \tag{S.7}$$

with the so-called prior mass variable $X_{\{\mathcal{M}^k\}_{\mathcal{I}^k}} \in [0,1]$ and $L_{\{\mathcal{M}^k\}_{\mathcal{I}^k}}$ the model set related likelihood expressed as a function of the prior mass variable

$$\begin{aligned} &L_{\{\mathcal{M}^k\}_{\mathcal{I}^k}} : [0,1] \to [0,\infty) \quad \text{with} \\ &L_{\{\mathcal{M}^k\}_{\mathcal{I}^k}}(X_{\{\mathcal{M}^k\}_{\mathcal{I}^k}}) = \sup\{\lambda \in \mathrm{im}(\mathcal{L}_{\{\mathcal{M}^k\}_{\mathcal{I}^k}}) \mid \xi(\lambda) > X_{\{\mathcal{M}^k\}_{\mathcal{I}^k}}\} \end{aligned} \tag{S.8}$$

where $\mathrm{im}(\mathcal{L}_{\{\mathcal{M}^k\}_{\mathcal{I}^k}})$ consists of all likelihood values for any element of the augmented flux space, and ξ the so-called surviving prior-mass function

$$\begin{aligned} &\xi : [0,\infty) \to [0,1] \text{ with} \\ &\xi(\lambda) = \mu_{\{\mathcal{M}^k\}_{\mathcal{I}^k}}(\{(i, v_{\mathcal{M}_i^k}) \in \Omega_{\{\mathcal{M}^k\}_{\mathcal{I}^k}} \mid \mathcal{L}_{\{\mathcal{M}^k\}_{\mathcal{I}^k}}(i, v_{\mathcal{M}_i^k}) > \lambda\}) \end{aligned} \tag{S.9}$$

Eq. (S.7) holds in the general case of measurable bounded likelihoods [11], which is fulfilled for the case of ^{13}C-MFA.

S.2.4 TDNS for BMSA

This section describes TDNS for BMSA for Bayesian model averaging in ^{13}C-MFA. We refer to the original literature on (trans-dimensional) DNS for technical details [5, 2]. Here, we focus on how the two core results, model set-averaged flux posterior samples and model set evidence, are computed

1. an approximation to the 1-dimensional evidence integral Eq. (S.7), and
2. model set-averaged flux samples $v^{(t)}_{\{\mathcal{M}^k\}_{\mathcal{I}^k}}$ to approximate the posterior $p(v_{\{\mathcal{M}^k\}_{\mathcal{I}^k}} \mid \mathcal{D})$

The following section introduces the notation and definitions required to understand TDNS for BMSA, as described in the algorithm listing S.1. Finally, we briefly discuss parameter choices, and how to diagnose TDNS for BMSA runs.

S.2.4.1 Solving the evidence integral

To approximately solve Eq. (S.7), TDNS constructs a ladder of $J_{\max} \in \mathbb{N}$ likelihood levels

$$0 = L^{(1)}_{\{\mathcal{M}^k\}_{\mathcal{I}^k}} < \cdots < L^{(j)}_{\{\mathcal{M}^k\}_{\mathcal{I}^k}} < \cdots < L^{(J_{\max})}_{\{\mathcal{M}^k\}_{\mathcal{I}^k}} \tag{S.10}$$

and estimates the prior masses

$$1 = X^{(1)}_{\{\mathcal{M}^k\}_{\mathcal{I}^k}} > \cdots > X^{(j)}_{\{\mathcal{M}^k\}_{\mathcal{I}^k}} > \cdots > X^{(J_{\max})}_{\{\mathcal{M}^k\}_{\mathcal{I}^k}} \tag{S.11}$$

such that

$$L_{\{\mathcal{M}^k\}_{\mathcal{I}^k}}(X^{(j)}_{\{\mathcal{M}^k\}_{\mathcal{I}^k}}) = L^{(j)}_{\{\mathcal{M}^k\}_{\mathcal{I}^k}}, \forall j \tag{S.12}$$

For each single model $\mathcal{M}^k_i$, the joint prior over its fluxes in view of the considered model set $\{\mathcal{M}^k\}_{\mathcal{I}^k}$ is given by

$$p(i, v_{\mathcal{M}^k_i}) = p(v_{\mathcal{M}^k_i} \mid \mathcal{M}^k_i) \cdot p(\mathcal{M}^k_i \mid \{\mathcal{M}^k\}_{\mathcal{I}^k}) \tag{S.13}$$

With this, we formulate the likelihood constrained prior probability for each model-flux combination given the j-th likelihood level as the prior-mass-weighted joint probability

$$p(i, v_{\mathcal{M}^k_i} \mid j) = \begin{cases} \frac{p(i, v_{\mathcal{M}^k_i})}{X^{(j)}_{\{\mathcal{M}^k\}_{\mathcal{I}^k}}}, & \text{if } \mathcal{L}_{\{\mathcal{M}^k\}_{\mathcal{I}^k}}(i, v_{\mathcal{M}^k_i}) > L^{(j)}_{\{\mathcal{M}^k\}_{\mathcal{I}^k}} \\ 0, & \text{otherwise} \end{cases} \tag{S.14}$$

The likelihood levels in Eq. (S.10) are constructed iteratively. Let J be the number of likelihood levels created so far. Following [5], we choose the probability of level j given the number of so far created levels J as follows

$$p_J(j) \propto \begin{cases} \exp\left(\frac{j-J}{\Lambda}\right), & \text{if } J < J_{\max} \\ \frac{1}{J_{\max}}, & \text{if } J = J_{\max} \end{cases} \tag{S.15}$$

with

$$\sum_{j=1}^{J} p_J(j) = 1, \forall J \tag{S.16}$$

and the back-tracking parameter $\Lambda \in \mathbb{R}^+$, controlling the width of $p_J(j)$.

We construct the likelihood level $L^{(j)}_{\{\mathcal{M}^k\}_{\mathcal{I}^k}}$ by alternately sampling the ladder-step j according to the probability $p_J(j)$ using Metropolis-Hastings and sampling model-flux combinations $(i, v_{\mathcal{M}^k_i})$ according to $p(i, v_{\mathcal{M}^k_i} \mid j)$ using RJMCMC. This alternate sampling produces samples (indicated by superscript (n))

$$j^{(n)} \sim p_J(j) \quad \text{and} \quad (i^{(n)}, v^{(n)}_{\mathcal{M}^k_i}) \sim p(i, v_{\mathcal{M}^k_i} | j^{(n)}) \tag{S.17}$$

Specifically, the likelihood level $L^{(j)}_{\{\mathcal{M}^k\}_{\mathcal{I}^k}}$ is created after sampling a set of samples with likelihood higher than the previous level $L^{(j-1)}_{\{\mathcal{M}^k\}_{\mathcal{I}^k}}$ and setting $L^{(j)}_{\{\mathcal{M}^k\}_{\mathcal{I}^k}}$ to the $(1-\alpha)$-quantile of the likelihoods of the samples, where $\alpha \in (0,1)$ is the so-called "compression factor". After the likelihood level $L^{(j)}_{\{\mathcal{M}^k\}_{\mathcal{I}^k}}$ has been constructed, the prior mass for that level $X^{(j)}_{\{\mathcal{M}^k\}_{\mathcal{I}^k}}$ is estimated according to nested sampling theory [1]:

$$X^{(j)}_{\{\mathcal{M}^k_i\}_{\mathcal{I}^k}} = \alpha \cdot X^{(j-1)}_{\{\mathcal{M}^k_i\}_{\mathcal{I}^k}} \tag{S.18}$$

Finally, with $N_{\max}$ samples and $J_{\max}$ levels generated, the trapezoidal rule is used to approximate the model evidence

$$p(\mathcal{D} \mid \{\mathcal{M}^k\}_{\mathcal{I}^k}) \approx \underbrace{\sum_{n=1}^{N_{\max}} \mathcal{L}_{\{\mathcal{M}^k\}_{\mathcal{I}^k}}(i^{(n)}, v^{(n)}_{\mathcal{M}^k_i}) \cdot \Delta\mathcal{X}^{(n)}_{\{\mathcal{M}^k\}_{\mathcal{I}^k}}}_{\hat{p}_{\mathcal{D}\mid\{\mathcal{M}^k\}_{\mathcal{I}^k}}} \tag{S.19}$$

with $\Delta\mathcal{X}^{(n)}_{\{\mathcal{M}^k\}_{\mathcal{I}^k}}$ the trapezoidal interval width for the n-th sample. Specifically, each augmented flux sample $(i^{(n)}, v^{(n)}_{\mathcal{M}^k_i})$ is assigned a prior-mass coordinate, denoted by $\mathcal{X}^{(n)}_{\{\mathcal{M}^k\}_{\mathcal{I}^k}}$, such that those with a higher likelihood are assigned a lower prior mass. If multiple samples evaluate to the same likelihood (i.e., "likelihood plateaus"), assigning prior masses to samples requires additional information [6, 11]. Here we rely on so-called "likelihood tie-breakers" [3], which resolve the issue of likelihood plateaus [6].

The prior-mass coordinate $\mathcal{X}^{(n)}_{\{\mathcal{M}^k\}_{\mathcal{I}^k}}$ is estimated by sampling uniformly from the interval $I_{j^{(n)}}$ given by

$$I_{j^{(n)}} = \begin{cases} \left(X^{(j^{(n)}+1)}_{\{\mathcal{M}^k\}_{\mathcal{I}^k}},\, X^{(j^{(n)})}_{\{\mathcal{M}^k\}_{\mathcal{I}^k}}\right], & \text{if } j^{(n)} < J_{\max} \\ \left[0,\, X^{(j^{(n)})}_{\{\mathcal{M}^k\}_{\mathcal{I}^k}}\right] & \text{if } j^{(n)} = J_{\max} \end{cases} \tag{S.20}$$

With that, the trapezoidal interval width $\mathcal{X}^{(n)}_{\{\mathcal{M}^k\}_{\mathcal{I}^k}}$ for the n-th sample is given by

$$\Delta\mathcal{X}^{(n)}_{\{\mathcal{M}^k\}_{\mathcal{I}^k}} = \frac{1}{2}\left(\mathcal{X}^{(n_+)}_{\{\mathcal{M}^k\}_{\mathcal{I}^k}} - \mathcal{X}^{(n_-)}_{\{\mathcal{M}^k\}_{\mathcal{I}^k}}\right) \tag{S.21}$$

with

$$\mathcal{X}^{(n_-)}_{\{\mathcal{M}^k\}_{\mathcal{I}^k}} = \max\left(\{0\} \cup \left\{\mathcal{X}^{(m)}_{\{\mathcal{M}^k\}_{\mathcal{I}^k}} \mid m \in \{1, \cdots, N_{\max}\},\, \mathcal{X}^{(m)}_{\{\mathcal{M}^k\}_{\mathcal{I}^k}} < \mathcal{X}^{(n)}_{\{\mathcal{M}^k\}_{\mathcal{I}^k}}\right\}\right) \tag{S.22}$$

and

$$\mathcal{X}^{(n_+)}_{\{\mathcal{M}^k\}_{\mathcal{I}^k}} = \min\left(\{1\} \cup \left\{\mathcal{X}^{(m)}_{\{\mathcal{M}^k\}_{\mathcal{I}^k}} \mid m \in \{1, \cdots, N_{\max}\},\, \mathcal{X}^{(m)}_{\{\mathcal{M}^k\}_{\mathcal{I}^k}} > \mathcal{X}^{(n)}_{\{\mathcal{M}^k\}_{\mathcal{I}^k}}\right\}\right) \tag{S.23}$$

where the 0 and 1 are added to the sets to deal with the two samples that have only one neighboring sample in prior mass space.

S.2.4.2 Posterior model set-averaged flux samples

With the estimated evidence in Eq. (S.19), normalized posterior weights $\eta^{(n)} \in [0,1]$ are computed for each sample $(i^{(n)}, v^{(n)}_{\mathcal{M}^k_i})$

$$\eta^{(n)} = \frac{\mathcal{L}_{\{\mathcal{M}^k\}_{\mathcal{I}^k}}(i^{(n)}, v^{(n)}_{\mathcal{M}^k_i}) \cdot \Delta\mathcal{X}^{(n)}_{\{\mathcal{M}^k\}_{\mathcal{I}^k}}}{\hat{p}_{\mathcal{D}\mid\{\mathcal{M}^k\}_{\mathcal{I}^k}}} \tag{S.24}$$

To obtain the final T posterior augmented flux samples, the samples $(i^{(n)}, v_{\mathcal{M}^k_i}{}^{(n)})$ are resampled according to their posterior weights $\eta^{(n)}$, such that the relative frequency of each point in the resampled set approximately represents its posterior weight. The model set-averaged fluxes $v_{\{\mathcal{M}^k\}_{\mathcal{I}^k}}$ are obtained by marginalizing over the i-component of the posterior augmented flux samples, where the i-component represents the model index of the posterior augmented flux samples. Furthermore, by counting the samples that point to a specific model

$\mathcal{M}_i^k$, the approximate posterior probability of this model is obtained given the considered model set. In the main text, for this we use the notation

$$p(\mathcal{M}_i^k|\{\mathcal{M}^k\}_{\mathcal{I}^k},\mathcal{D}) \approx \frac{\#\text{samples}(\mathcal{M}_i^k)}{\#\text{samples}(\{\mathcal{M}^k\}_{\mathcal{I}^k})},\ \forall i,k \tag{S.25}$$

where #samples($\mathcal{M}_i^k$) denotes how often a model $\mathcal{M}_i^k$ was sampled and #samples($\{\mathcal{M}^k\}_{\mathcal{I}^k}$) is the total number of posterior samples drawn for the model set k.

S.2.4.3 The TDNS for BMSA algorithm

Algorithm S.1 TDNS for BMSA

Input:
Model set $\{\mathcal{M}^k\}_{\mathcal{I}^k}$
Priors for fluxes $p(v_{\mathcal{M}_i^k} \mid \mathcal{M}_i^k)$ and all models constituting the model set $p(\mathcal{M}_i^k \mid \{\mathcal{M}^k\}_{\mathcal{I}^k})$
Maximum number of samples to draw $N_{\max}$
Maximum number of likelihood levels $J_{\max}$
Level interval size l_{new}
Back-tracking parameter Λ
Compression factor $\alpha \in (0, 1)$
Output:
Posterior model set-averaged flux samples $\{v^{(t)}_{\{\mathcal{M}^k\}_{\mathcal{I}^k}}\}_{t=1}^{T}$ with $v^{(t)}_{\{\mathcal{M}^k\}_{\mathcal{I}^k}} \sim p(v_{\{\mathcal{M}^k\}_{\mathcal{I}^k}} \mid \mathcal{D})$
Evidence estimate $\hat{p}_{\mathcal{D}|\{\mathcal{M}^k\}_{\mathcal{I}^k}}$
Procedure:

```
n ← 0
J ← 1                                                          ▷ Set likelihood level counter
L^(J)_{M^k}_I^k ← 0                                            ▷ Create initial likelihood level
X^(J)_{M^k}_I^k ← 1                                            ▷ Set initial prior mass
while J ≤ J_max do
    L_seen ← ∅                                                 ▷ Initialize store for seen likelihoods
    l ← 0
    while l < l_new do
        n ← n + 1                                              ▷ Increment number of samples
        Metropolis-Hastings sample j^(n) ~ p_J(j)              ▷ Level move; cf. Eq. (S.15)
        RJMCMC sample (i^(n), v^(n)_{M_i^k}) ~ p(i, v_{M_i^k} | j^(n))   ▷ Parameter move; cf. Eq. (S.14)
        if n = N_max then
            Go to Evaluation
        end if
        if ℒ_{M^k}_I^k(i^(n), v^(n)_{M_i^k}) > L^(J)_{M^k}_I^k then
            l ← l + 1                                          ▷ Increment number of likelihoods in next level interval
            L_seen ← L_seen ∪ {ℒ_{M^k}_I^k(i^(n), v^(n)_{M_i^k})}   ▷ Store likelihood of n-th sample
        end if
    end while
    ▷ Create next likelihood level based on seen likelihoods
    J ← J + 1                                                  ▷ Increment likelihood level counter
    L^(J)_{M_i^k}_I^k ← Quantile(L_seen, 1 − α)
    X^(J)_{M^k}_I^k ← α · X^(J−1)_{M^k}_I^k                    ▷ Set prior mass by compression of forerunner level
end while
while n < N_max do
    n ← n + 1                                                  ▷ Increment number of samples
    Metropolis-Hastings sample j^(n) ~ p_J(j)                  ▷ Level move; see Eq. (S.15)
    RJMCMC sample (i^(n), v^(n)_{M_i^k}) ~ p(i, v_{M_i^k} | j^(n))   ▷ Parameter move; cf. Eq. (S.14)
end while
```

Evaluation:
1) Estimate the prior-mass interval widths $\{\Delta\mathcal{X}^{(n)}_{\{\mathcal{M}^k\}_{\mathcal{I}^k}}\}_{n=1}^{N_{\max}}$ according to Eq. (S.21)
2) Use $\{\Delta\mathcal{X}^{(n)}_{\{\mathcal{M}^k\}_{\mathcal{I}^k}}\}_{n=1}^{N_{\max}}$ to obtain evidence estimate $\hat{p}_{\mathcal{D}|\{\mathcal{M}^k\}_{\mathcal{I}^k}}$ according to Eq. (S.19)
3) Reweigh augmented flux space samples $\{(i^{(n)}, v^{(n)}_{\mathcal{M}_i^k})\}_{n=1}^{N_{\max}}$ according to Eq. (S.24) and marginalize out i-component so that T model averaged flux posterior samples $\{v^{(t)}_{\{\mathcal{M}^k\}_{\mathcal{I}^k}}\}_{t=1}^{T}$ are obtained

S.2.4.4 Parameterization, diagnostics, and reproducibility

TDNS for BMSA intakes a set of parameters that control the sampling processes, allowing the algorithm to be adapted to the difficulty of the given ^{13}C-MFA problem.
The problem-specific parameters are $J_{\max}$, l_{new}, and Λ. Suitable values for these parameters are determined using tuning runs. Generally, higher values of these three parameters provide more robust results, but also increase computational costs. We found that more levels are required when the posterior is concentrated in a small region compared to the prior. This finding has also been reported previously [7]. Higher values of Λ and l_{new} are beneficial, when the likelihood has pronounced multi-modality and the MCMC sampling is slow to explore the augmented flux space, see for example [3].
Two further parameters are less closely tied to the problem characteristic in the sense, that they do not require tuning runs: the compression factor α and the maximum number of samples to be drawn, $N_{\max}$. The default compression factor is set to $\alpha = e^{-1}$ [3]. The $N_{\max}$ parameter is flexible and adaptable because sampling runs can always be continued if more samples are required. For example, it is necessary to continue sampling if the desired $J_{\max}$ levels have not yet been reached or if the information contained by the T posterior samples should be further increased. The ability to extend runs is a property that sets DNS and TDNS apart from other nested sampling algorithms. Notably, problems with similar concentration of posterior mass relative to the prior exhibit comparable computational cost [7], enabling transfer of TDNS parameterizations across related inference tasks and thereby reducing the need for extensive tuning. We have previously tested TDNS configurations for different data sets, providing reference points for tuning BMSA [9].
All parameter values used for all case studies are found in the respective computational scripts. The specific parameter values, which we used in our *E. coli* case studies are also reported in Section S.4.5.

For diagnostics, we followed the recommendation in [3] and checked that the increase in likelihood values for decreasing $X_{\{\mathcal{M}^k\}_{\mathcal{I}^k}}$ slowed down, resulting in a well-visible peak in the associated posterior weights of the samples (Eq. (S.24)) as a function of $X_{\{\mathcal{M}^k\}_{\mathcal{I}^k}}$; see [3] for more details and Section S.4.5.

We recommend to rerun TDNS for BMSA several times with different random seeds for new problems. Since there are no explicit starting points in TDNS (uniform sampling, i.e., prior sampling mixes extremely well on convex polytopes [8]), changing the random seed ensures that the algorithm uses different samples to explore flux and model spaces. In addition, multiple runs improve evidence estimation.

S.3 *Triangulus*: Additional information

S.3.1 Prior specification

k	i	DOF	$p(\{\mathcal{M}^k\}_{\mathcal{I}^k})$	$p(\mathcal{M}_i^k \mid \{\mathcal{M}^k\}_{\mathcal{I}^k})$	$p(\mathcal{M}_i^k)$	$p(v \mid \mathcal{M}_i^k)$
1	1	1	1/3	1/2	1/6	$\begin{cases} 1/1000, & \text{if } v \in \mathcal{P}_{\mathcal{M}_1^1} \\ 0, & \text{otherwise} \end{cases}$
1	2	2		1/2	1/6	$\begin{cases} 1/1000^2, & \text{if } v \in \mathcal{P}_{\mathcal{M}_2^1} \\ 0, & \text{otherwise} \end{cases}$
2	1	2	1/3	1/2	1/6	$\begin{cases} 2/1000^2, & \text{if } v \in \mathcal{P}_{\mathcal{M}_1^2} \\ 0, & \text{otherwise} \end{cases}$
2	2	3		1/2	1/6	$\begin{cases} 2/1000^3, & \text{if } v \in \mathcal{P}_{\mathcal{M}_2^2} \\ 0, & \text{otherwise} \end{cases}$
3	1	3	1/3	1/2	1/6	$\begin{cases} 2/(5 \cdot 1000^2), & \text{if } v \in \mathcal{P}_{\mathcal{M}_1^3} \\ 0, & \text{otherwise} \end{cases}$
3	2	4		1/2	1/6	$\begin{cases} 2/(5 \cdot 1000^3), & \text{if } v \in \mathcal{P}_{\mathcal{M}_2^3} \\ 0, & \text{otherwise} \end{cases}$

Table S.1: Model and flux priors for the *Triangulus* study. As the DOF increase, the flux priors are effectively diffused across the larger flux spaces. For $k = 3$, the added flux going out of metabolite `D` is constrained to $[0, 5]$, meaning the polytope volume scales with $5 \cdot 1000^{\#\text{DOF-1}}$, rather than $1000^{\#\text{DOF}}$.

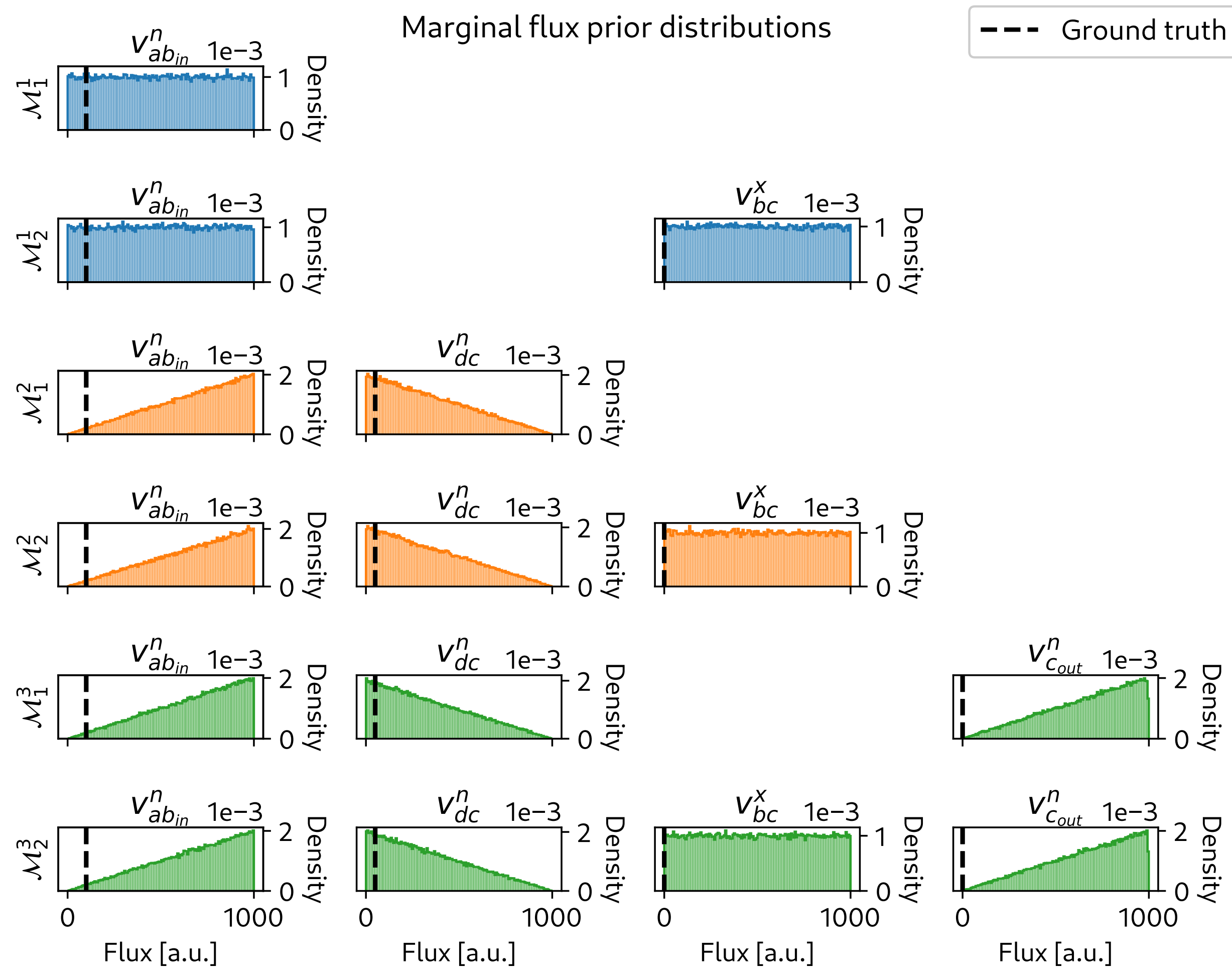

Figure S.2: Marginal flux priors for the *Triangulus* case study. Each row corresponds to a different model and shows all its fluxes. $\mathcal{M}_1^2$ is the data generating *Triangulus* model with ground truth fluxes indicated by dashed lines. For the models of the green model set, zero values of v^n_{dout} and v^x_{bc} collapse the models to the (nested) *Triangulus* model.

S.3.2 Marginal posteriors

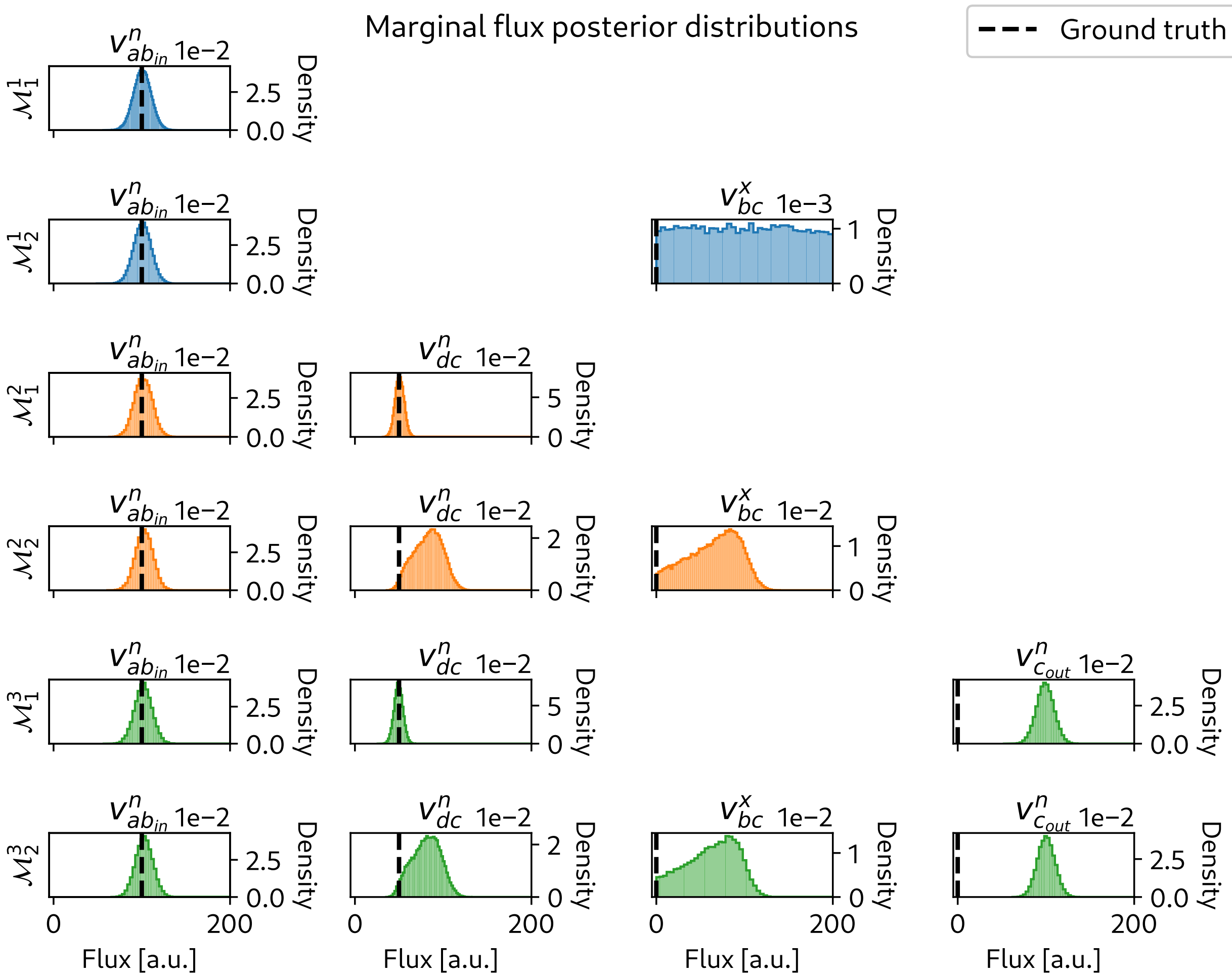

Figure S.3: Single-model flux posteriors for the *Triangulus* case study. Each row corresponds to a different model and shows all of its fluxes. The ground truth fluxes are indicated by dashed lines. Only the ground truth model ($\mathcal{M}_1^2$, third row) accurately recovers all the fluxes. The more complex models exhibit biases for the internal fluxes. The models in the blue model set are unable to explain the data due to the way they are constructed.

S.3.3 TDNS diagnostics

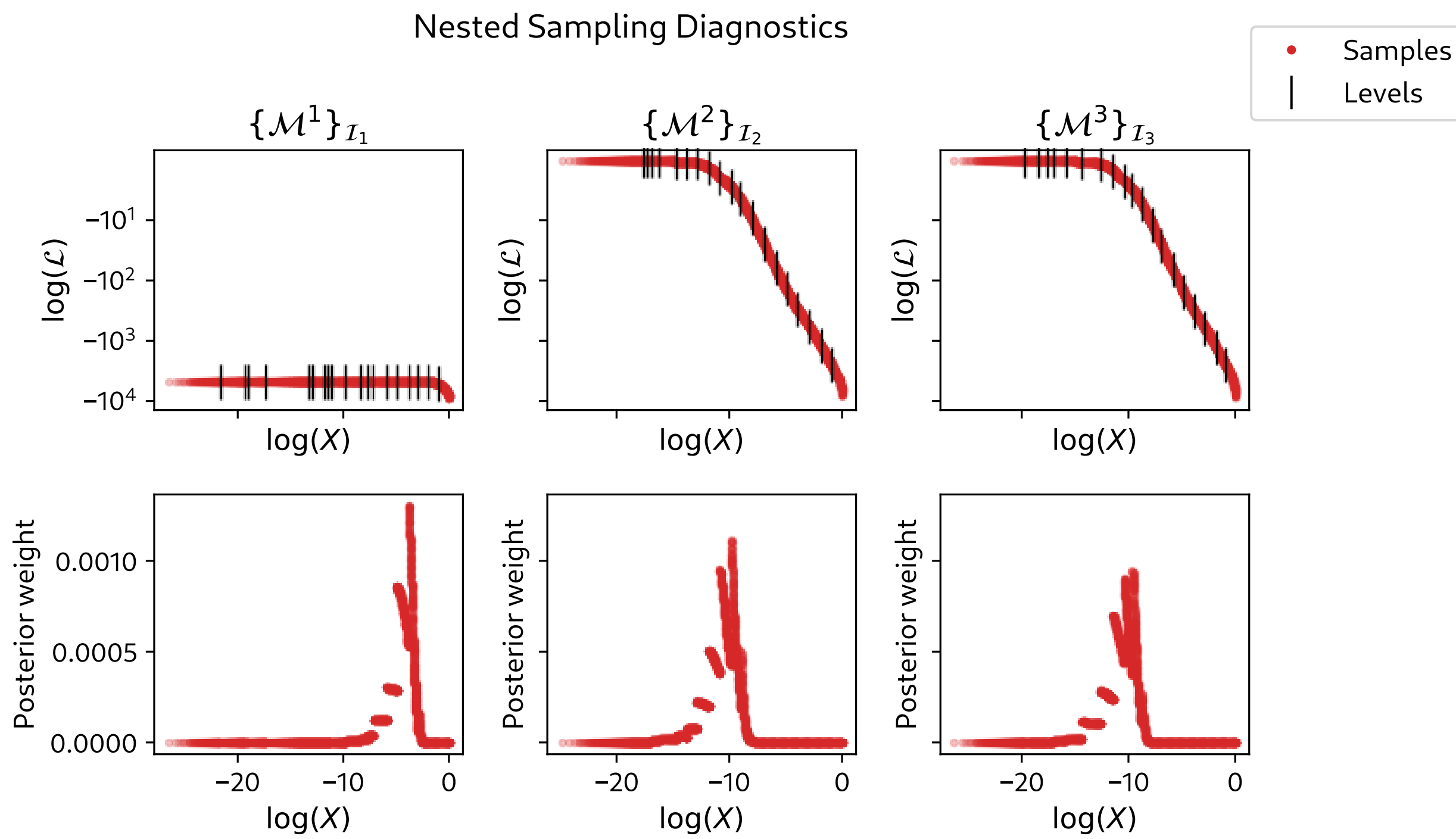

Figure S.4: Diagnostics of the TDNS runs for the *Triangulus* case study. Each column corresponds to one model subset. The first row shows the log-likelihood over the prior mass $X_{\{\mathcal{M}^k\}_{\mathcal{I}^k}}$. The increase in likelihood levels off once sufficient prior mass has been accumulated. In the bottom row, sharp peaks in the posterior weight indicate that the typical set has been identified. There is no posterior weight for very small prior masses $\log(X_{\{\mathcal{M}^k\}_{\mathcal{I}^k}})$. BMSA took 30.7 seconds on a laptop (Intel Core i7-13700H) with each model set $\{\mathcal{M}^k\}_{\mathcal{I}^k}$ run sequentially.

S.4 *Escherichia coli* use case: Additional information

S.4.1 Reference model and flux map

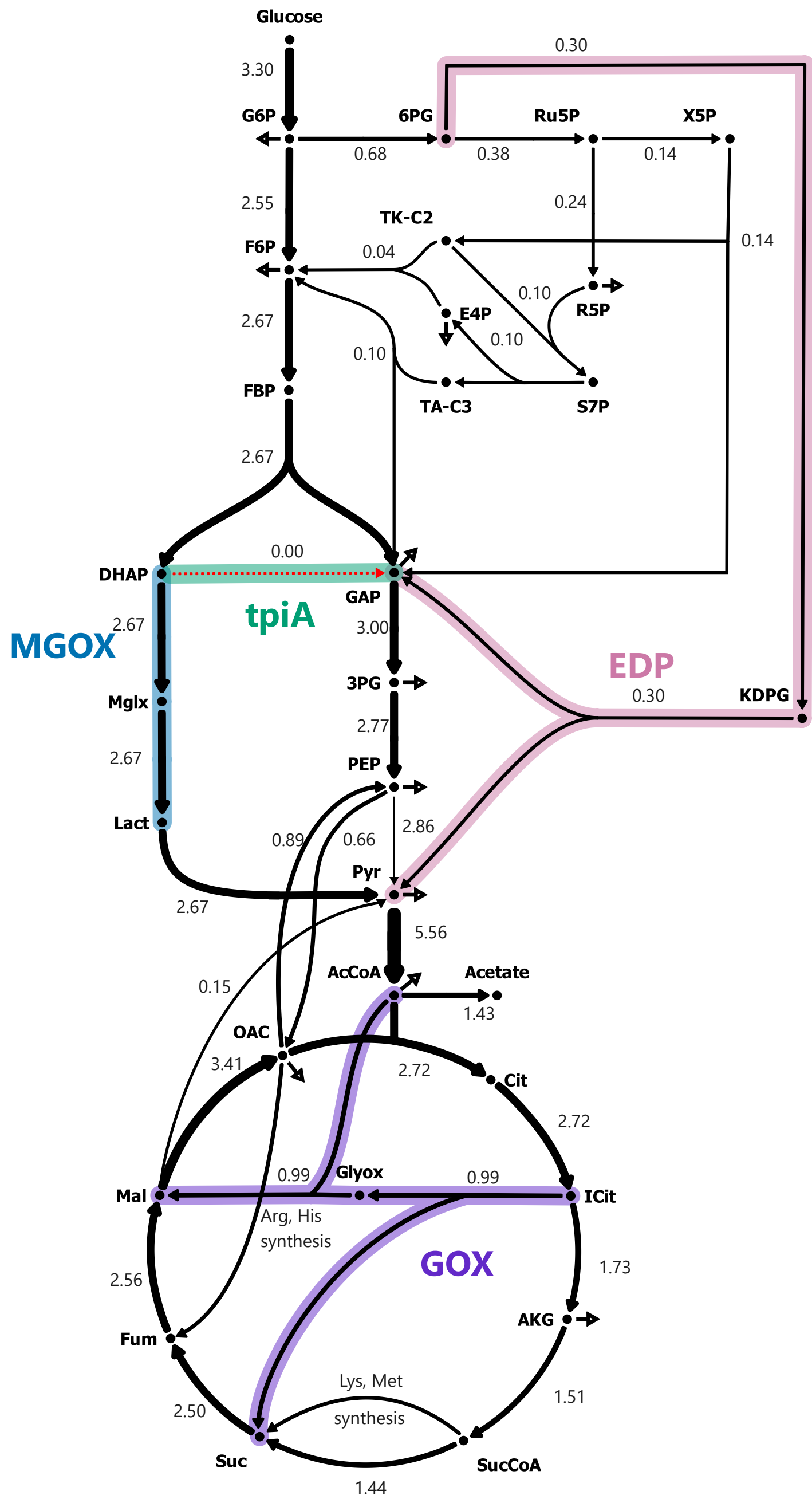

Figure S.5: Reference metabolic map for the *Escherichia coli* case study. The latent pathways with uncertain metabolic activity (GOX - glyoxylate shunt, MGOX - methylglyoxal pathway, EDP - Entner-Doudoroff pathway), as well as the TPI pathway with the triosephosphate isomerase gene knocked out, are highlighted. Values give net fluxes in mmol g_{CDW}^{-1} h^{-1}. The reference model has 11 (independent) net fluxes and 23 exchange fluxes, resulting in 34 DOF.

S.4.2 Model subsets

k	reactions sets	DOF range	cardinality	FluxML filename
1	EDP-GOX-MGOX	11-44	2^{33}	`e_coli_+EDP+GOX+MGOX-TPI.fml`
2	GOX-MGOX	10-41	2^{31}	`e_coli_-EDP+GOX+MGOX-TPI.fml`
3	GOX-TPI	10-40	2^{30}	`e_coli_-EDP+GOX-MGOX+TPI.fml`
4	EDP-GOX-TPI	11-43	2^{32}	`e_coli_+EDP+GOX-MGOX+TPI.fml`
5	GOX-MGOX-TPI	11-43	2^{32}	`e_coli_-EDP+GOX+MGOX+TPI.fml`
6	EDP-GOX-MGOX-TPI	12-46	2^{34}	`e_coli_+EDP+GOX+MGOX+TPI.fml`
7	EDP-MGOX	10-41	2^{31}	`e_coli_+EDP-GOX+MGOX-TPI.fml`
8	MGOX	9-38	2^{29}	`e_coli_-EDP-GOX+MGOX-TPI.fml`
9	TPI	9-37	2^{28}	`e_coli_-EDP-GOX-MGOX+TPI.fml`
10	EDP-TPI	10-40	2^{30}	`e_coli_+EDP-GOX-MGOX+TPI.fml`
11	MGOX-TPI	10-40	2^{30}	`e_coli_-EDP-GOX+MGOX+TPI.fml`
12	EDP-MGOX-TPI	11-43	2^{32}	`e_coli_+EDP-GOX+MGOX+TPI.fml`

Table S.2: Model subsets for the *Escherichia coli* case study. Out of the 2^4=16 combinations of the four latent pathways with uncertain metabolic activity, four combinations are stoichiometrically impossible because they contain a dead-end metabolite (i.e. DHAP), which renders either TPI, MGOX or both active. Therefore, twelve stoichiometrically model sets are valid. The smallest model set $\{\mathcal{M}^9\}_{\mathcal{I}^9}$ (TPI) contains 268,435,456 unique model structures. The most comprehensive set $\{\mathcal{M}^6\}_{\mathcal{I}^6}$ contains 17,179,869,184 different model variants and is 64 times larger. Together, all model sets contain 46,976,204,800 models. The DOF range over all models is 9-46. Each model subset is based on a distinct reaction set, specified in a single FluxML file.

S.4.3 Prior specification

For our studies, we use the following setting for the models

$$p(\{\mathcal{M}^k\}_{\mathcal{I}^k}) = \tfrac{1}{12} \text{ and } p(\mathcal{M}_i^k \mid \{\mathcal{M}^k\}_{\mathcal{I}^k}) = \tfrac{1}{|\{\mathcal{M}^k\}_{\mathcal{I}^k}\}|}. \tag{S.26}$$

With that, the model priors are

$$p(\mathcal{M}_i^k) = \tfrac{1}{12\cdot|\{\mathcal{M}^k\}_{\mathcal{I}^k}|} \tag{S.27}$$

For the fluxes, we use a polytope-constrained uniform prior with additional boxed constraints, i.e. 100 times the uptake rate in both directions

$$-330\,\text{mmol/g}_{\text{CDW}}\text{/hour} \leq v_{\mathcal{M}_i^k} \leq 330\,\text{mmol/g}_{\text{CDW}}\text{/hour}, \, \forall i \in \mathcal{I}^k \tag{S.28}$$

where the vector inequality is to be understood component-wise.

S.4.4 Marginal posteriors

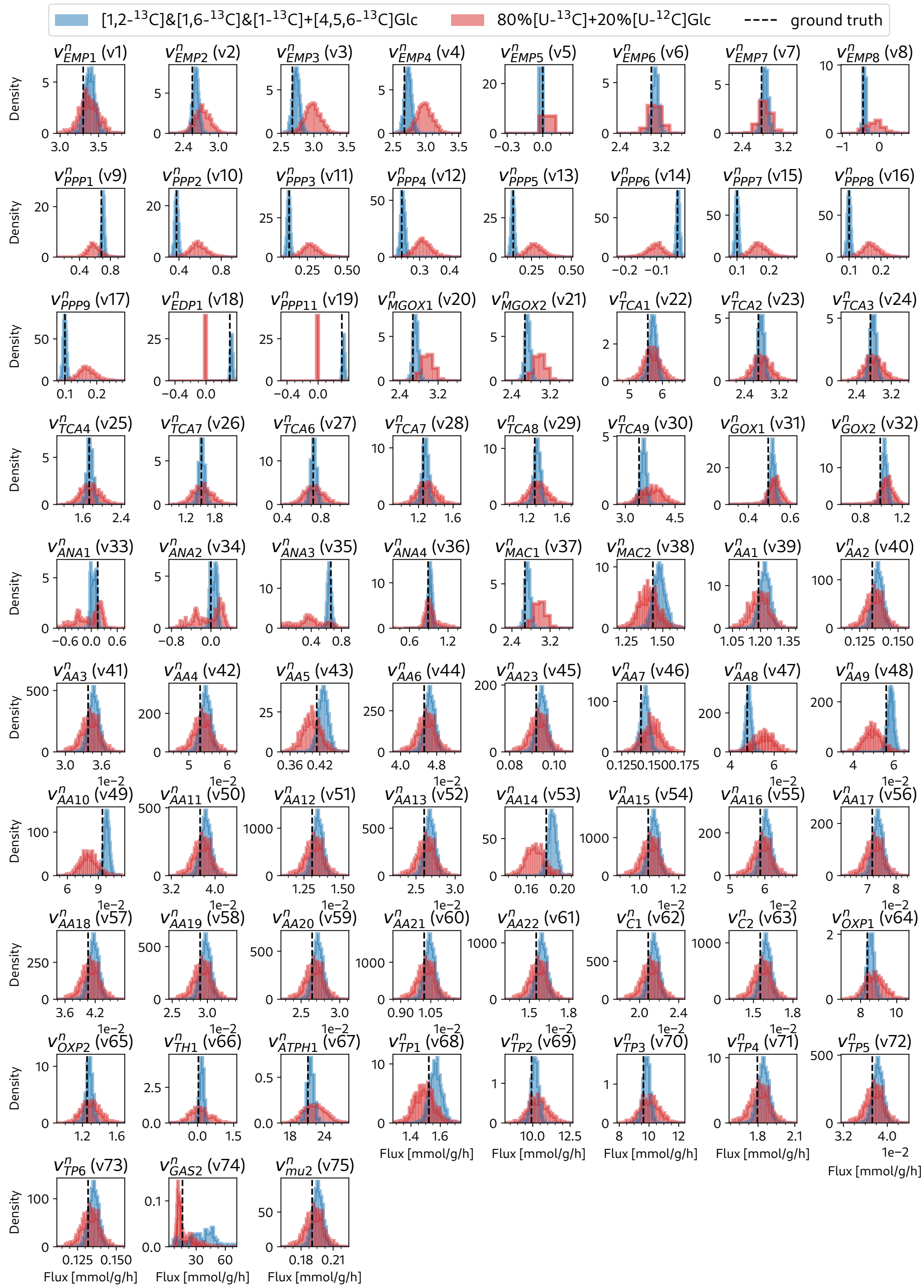

Figure S.6: Marginal full posteriors for all net fluxes contained in at least one of the models in the full model set $\{\mathcal{M}^k\}_\mathcal{K}$. Posteriors are shown for both the single-ILE evaluation and the multi-ILE evaluation. As expected, the marginal distributions are wider for the single dataset evaluation. The names of the fluxes refer to the naming convention used in the FluxML files, while the names in brackets refer to the original study in [10].

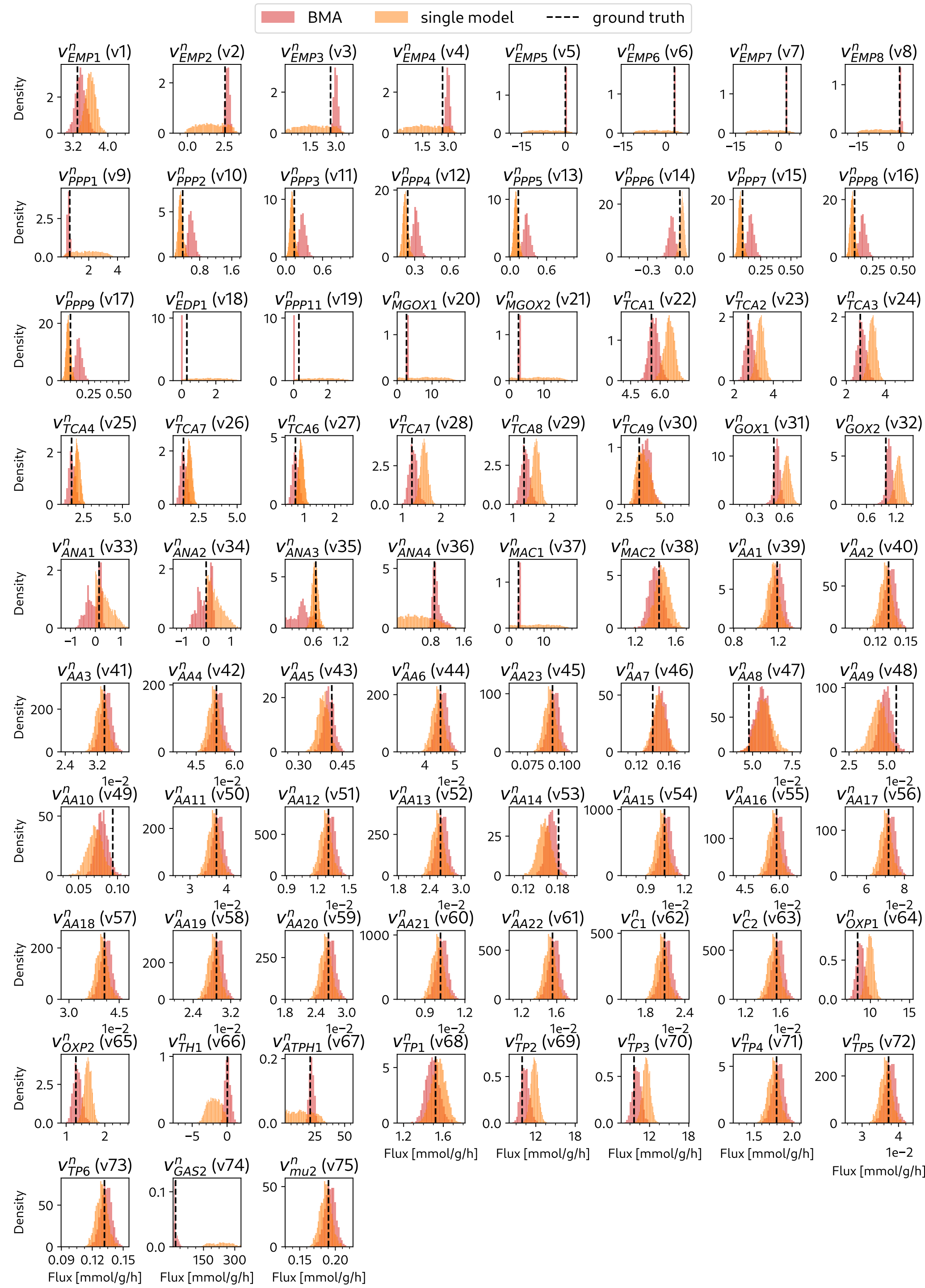

Figure S.7: Comparison of BMA-based inference using BMSA and the traditional single-model inference approach for the single-ILE evaluation. The marginal posteriors for all net fluxes are shown. For the traditional analysis, a super-model based on the original study by [10] was used. This model contains all latent pathways (EDP-GOX-MGOX-TPI) with 38 DOF (12 independent net fluxes and 26 exchange fluxes). Flux names refer to the naming convention used in the FluxML files, with names in brackets referring to those in the original study. Interestingly, the flux marginals for core fluxes in EMP and PPP are wider than for the single-model analysis compared to the BMA approach based on an extensive model set, indicating more uncertainty. This discrepancy arises because the data support a set of simpler models compared to the original model.

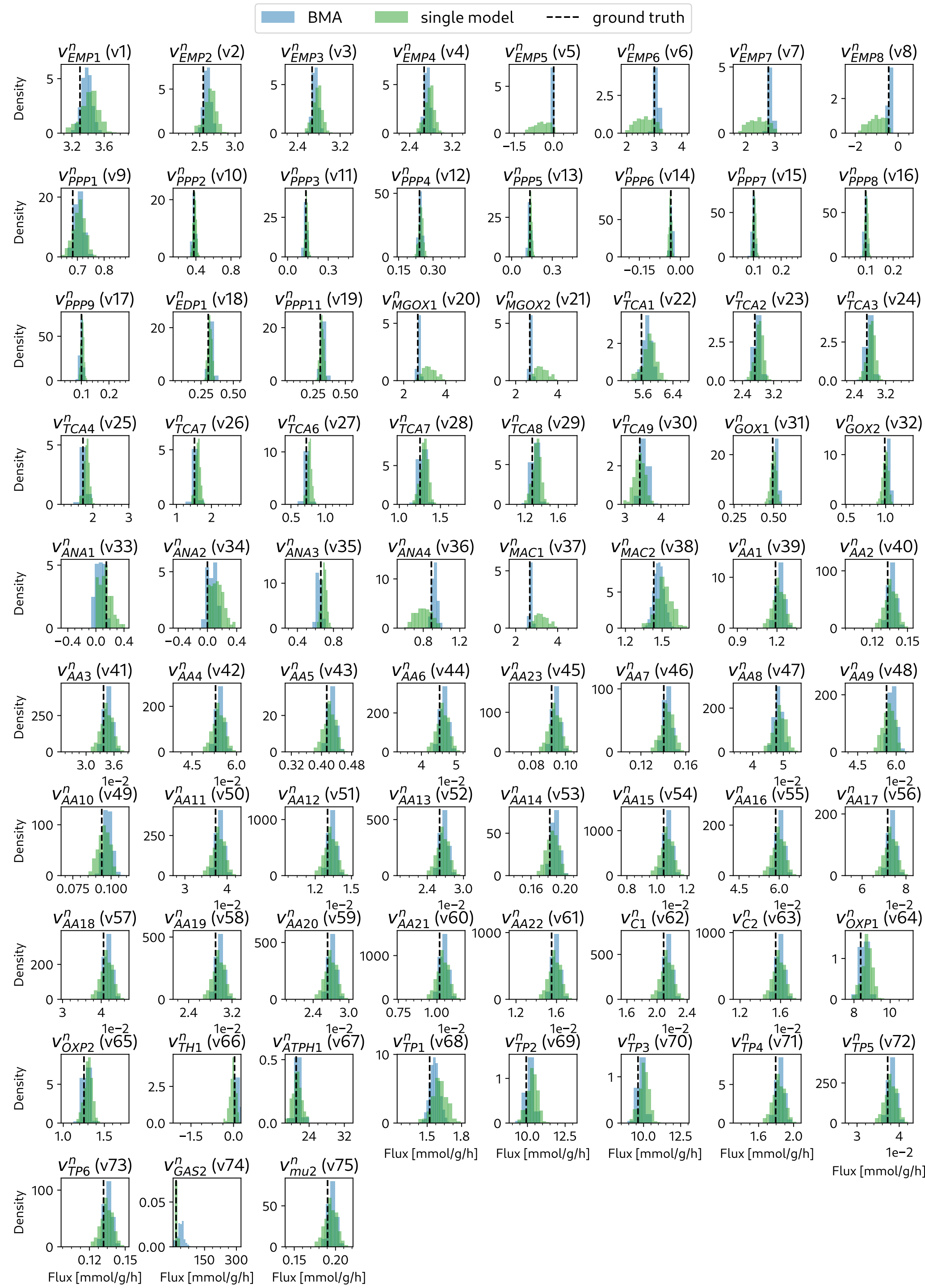

Figure S.8: Comparison of BMA-based inference generated using BMSA and the single-model inference approach for the multi-ILE evaluation. The marginal posteriors for all net fluxes are shown. For the single-model analysis, a super-model from the original study by [10] was used. This model contains all latent pathways (EDP-GOX-MGOX-TPI) and has 38 DOF (12 independent net fluxes and 26 exchange fluxes). Flux names refer to the naming convention used in the FluxML files, with names in brackets referring to those in the original study. Interestingly, the flux marginals for core fluxes in EMP and PPP are wider for the single-model analysis than for the BMA approach based on an extensive model set, indicating more uncertainty. This discrepancy is explained by the fact that BMA inference supports simpler models (effective model set), given the data, as compared to the original model. Notably, the differences between single-model and multi-model approaches are starkly reduced compared to the results of the single-ILE dataset evaluation shown in Figure S.7.

S.4.5 TDNS for BMSA parameters & diagnostics

In our experiments, we used the following TDNS for BMSA parameters for the single-ILE dataset evaluation

- $J_{\max} = 225$
- $\Lambda = 50$

and for the multi ILE dataset evaluation

- $J_{\max} = 375$
- $\Lambda = 80$

In both cases, l_{new} was set to 360,000 and 128 parallel RJMCMC chains were used.

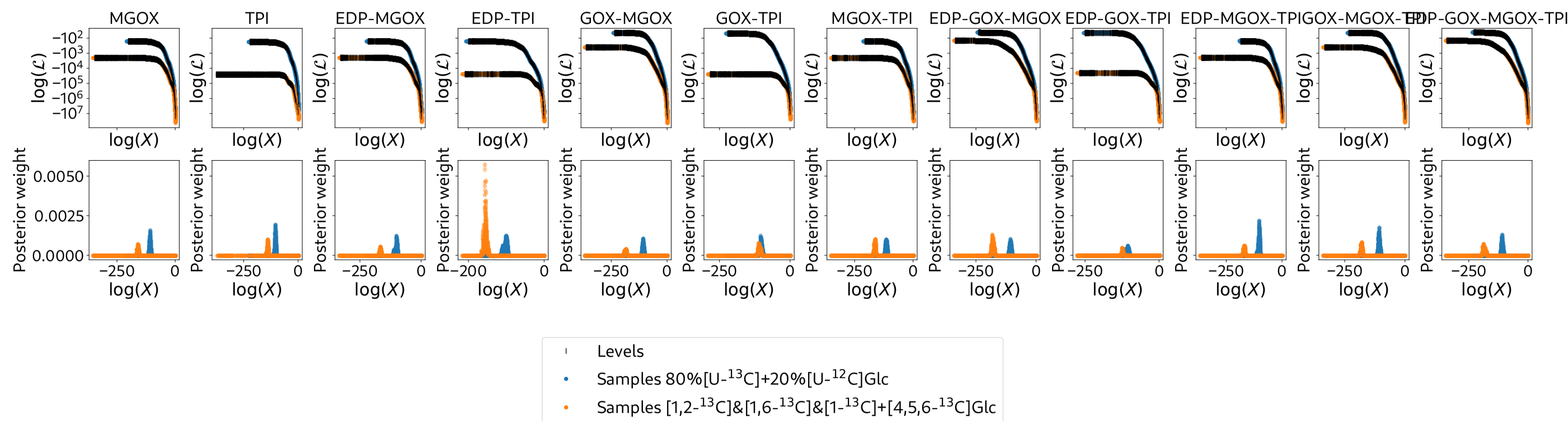

Figure S.9: Diagnostics of the TDNS for BMSA runs for *Escherichia coli*. Each column corresponds to one of the model subsets in Table S.2. In the upper row, the log-likelihood over the prior mass $X_{\{\mathcal{M}^k\}_{\mathcal{I}^k}}$ is shown. The increase in likelihood levels off as soon as sufficient prior mass has been accumulated. In the lower row, sharp peaks in the posterior weight indicate that the typical set has been identified. Notably, there is no posterior weight for small prior masses. The results of the single-ILE evaluation show a larger negative log-likelihood than the multi-ILE analysis, as the former contains only one-third of the measurements of the latter.

S.4.6 Reproducibility of results

To study the reproducibility of BMSA, the inferences were re-run with a different random seed for both, the single-ILE and multi-ILE cases. Figure S.10 shows the results for the two runs with the same reference fluxes shown as in main Figure 2. The activation probabilities for the bidirectional reactions in both runs are given in Table S.3. The two runs produced the same results, so we conclude that inferences computed by BMSA are reproducible.

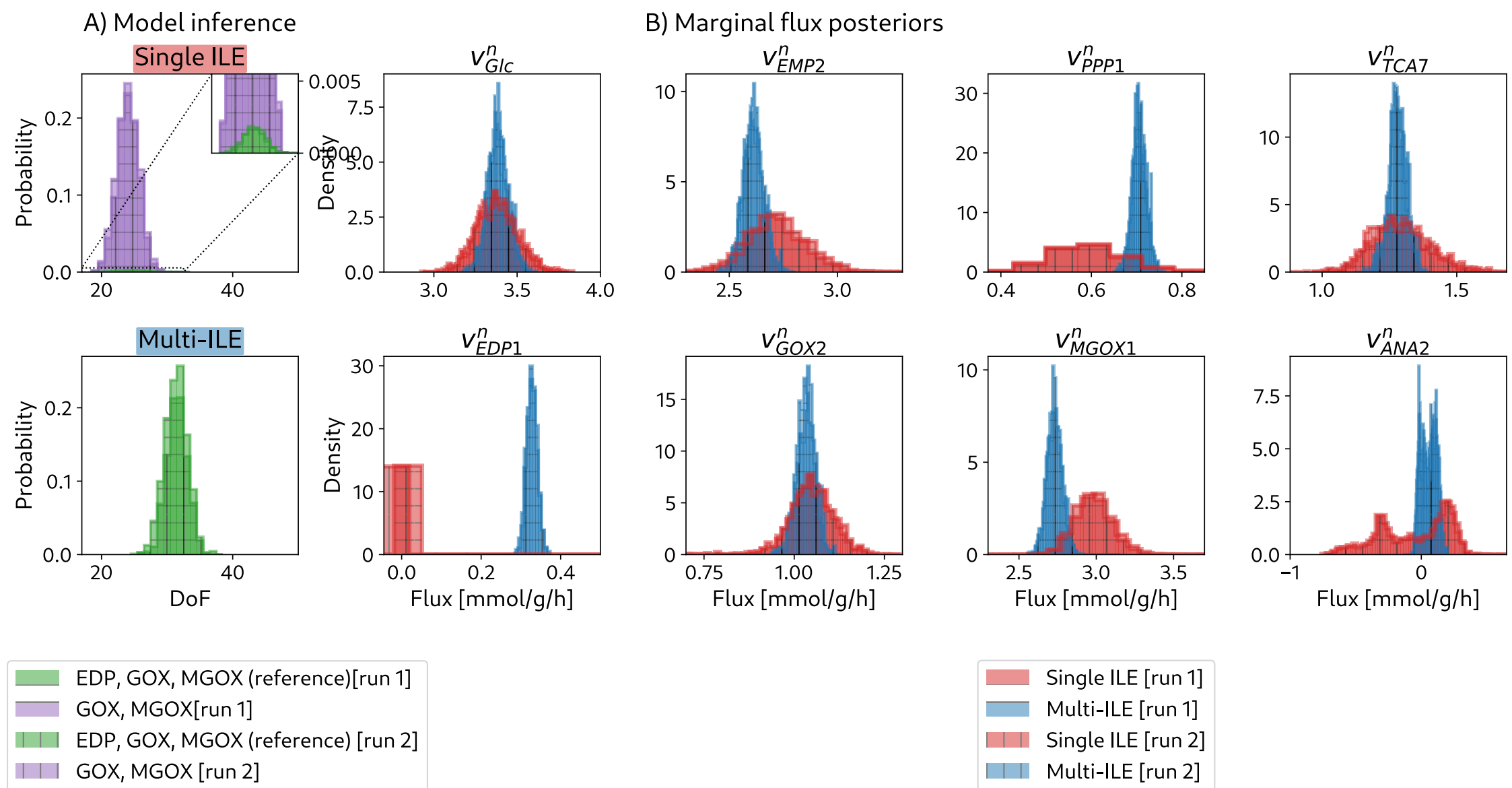

Figure S.10: Result summary for BMSA run 1 and 2 of the single and multi-ILE evaluation. Hatches were added to distinguish the runs while keeping the colors. The results of both runs are the same.

Reaction	Single ILE run1 Act. prob.	Single ILE run2 Act. prob.	Multi-ILE run1 Act. prob.	Multi-ILE run2 Act. prob.
AA8_v47	0.0000	0.0000	1.0000	1.0000
AA9_v48	0.0000	0.0000	0.0000	0.0000
ANA1_v33	0.0023	0.0000	0.0000	0.0000
ANA2_v34	0.0000	0.0004	0.0000	0.0000
EMP2_v2	1.0000	1.0000	1.0000	1.0000
EMP4_v4	0.5170	0.5093	0.5044	0.4583
EMP6_v6	0.9950	0.9946	1.0000	1.0000
EMP7_v7	1.0000	1.0000	1.0000	1.0000
EMP8_v8	0.1392	0.1442	1.0000	1.0000
GOX1_v31_2	0.0050	0.0131	0.0073	0.0273
GOX2_v32	0.0073	0.0116	0.0079	0.0038
MAC1_v37	0.4887	0.5189	0.4977	0.5106
MAC2_v38	0.5217	0.5197	0.4953	0.5258
MGOX2_v21	0.5190	0.4691	0.5196	0.5303
PPP10_v18	0	0	0.3160	0.3500
PPP11_v19	0	0	0.3531	0.3068
PPP3_v11	0.4207	0.4706	1.0000	1.0000
PPP4_v12	0.4737	0.4845	0.9997	0.9992
PPP5_v13	0.4254	0.4385	1.0000	1.0000
PPP6_v14	0.9970	0.9988	1.0000	1.0000
PPP7_v15	0.3298	0.3438	1.0000	1.0000
PPP8_v16	1.0000	1.0000	1.0000	1.0000
PPP9_v17	0.3371	0.3337	1.0000	1.0000
TCA1_v22	0.0010	0.0012	0.0020	0.0000
TCA2_v23	0.3804	0.4172	0.5044	0.5538
TCA3_v24	0.3781	0.3855	0.5061	0.4970
TCA4_v25	0.8101	0.7208	0.0070	0.0114
TCA6_v27_2	0.4880	0.5089	0.8072	0.8871
TCA7_v26	0.0027	0.0073	0.6290	0.7947
TCA7_v28_2	0.5143	0.4884	0.5366	0.6098
TCA8_v29_2	0.2525	0.3314	1.0000	1.0000
TCA9_v30	0.4004	0.3747	1.0000	1.0000
TH1_v66	0.4833	0.5043	0.5149	0.5091

Table S.3: Activation probability for the bidirectionality of all reactions for two single-ILE and two multi-ILE BMSA runs. The maximum discrepancy in probability for the reaction bidirectionalities between runs for the single-ILE evaluation is around 0.089 (TCA4_v25) and around 0.166 (TCA4_v26) for the multi-ILE case. Conversely, many probabilities are identical between runs. Overall, this result is within the expected variance of TDNS (particularly the RJMCMC part), and we can therefore conclude that our runs are reproducible with respect to the fluxes. The model set inferred for the single-ILE evaluation does not contain the EDP pathway, meaning we assign the value 0 to the activation probabilities to the bidirectionality of the EDP reactions PPP10_v18 and PPP11_v19.

Nomenclature

$\mathcal{D}$ Data

$\mathcal{I}$, $\mathcal{I}^k$ Finite index sets with running index i

$\mathcal{K} = \bigcup_{k=1}^{K} \mathcal{I}^k$ Finite index set consisting of the union of k index sets $\mathcal{I}^k$

$\mathcal{M}$ Single model in traditional ^{13}C-MFA

$\mathcal{M}_i$ Single model from model set $\{\mathcal{M}^k\}_{\mathcal{I}}$

$\mathcal{M}_i^k$ Single model from model subset $\{\mathcal{M}^k\}_{\mathcal{I}^k}$ or full model set $\{\mathcal{M}^k\}_{\mathcal{K}}$

$\mathcal{P}_{\mathcal{M}}$, $\mathcal{P}_{\mathcal{M}_i^k}$ Model-specific flux polytope

$\{\mathcal{M}^k\}_{\mathcal{I}^k}$ Model subset

$\{\mathcal{M}^k\}_{\mathcal{K}}$ Full model set

i', i'', k', k'' Sum variables

n_x Number of reactions with unknown bidirectionality in a single model $\mathcal{M}$

$p(\mathcal{D} \mid \mathcal{M}_i^k)$ Model evidence of single model $\mathcal{M}_i^k$

$p(\mathcal{D} \mid \{\mathcal{M}^k\}_{\mathcal{I}^k})$ Aggregated model evidence of all models in $\{\mathcal{M}^k\}_{\mathcal{I}^k}$

$p(\mathcal{D} \mid v_{\mathcal{M}_i^k})$ Model-specific likelihood

$p(\mathcal{M}_i^k \mid \{\mathcal{M}^k\}_{\mathcal{I}^k})$ Model prior in view of the model subset $\{\mathcal{M}^k\}_{\mathcal{I}^k}$

$p(\mathcal{M}_i^k \mid \{\mathcal{M}^k\}_{\mathcal{I}^k}, \mathcal{D})$ Single-model posterior probabilities in view of the model subset $\{\mathcal{M}^k\}_{\mathcal{I}^k}$

$p(\mathcal{M}_i^k \mid \{\mathcal{M}^k\}_{\mathcal{K}})$ Model prior in view of the full model set $\{\mathcal{M}^k\}_{\mathcal{K}}$

$p(\mathcal{M}_i^k \mid \{\mathcal{M}^k\}_{\mathcal{K}}, \mathcal{D})$ Single-model posterior probabilities in view of the full model set $\{\mathcal{M}^k\}_{\mathcal{K}}$

$p(\mathcal{M}_i^k \mid \{\mathcal{M}^k{}_{\mathcal{I}^k}\})$ Model prior in view of the model subset $\{\mathcal{M}^k\}_{\mathcal{I}^k}$

$p(\mathcal{M}_i^k)$ Model prior in view of the full model set $\{\mathcal{M}^k\}_{\mathcal{K}}$

$p(\{\mathcal{M}^k\}_{\mathcal{I}^k} \mid \mathcal{D})$ Model subset posterior probability

$p(\{\mathcal{M}^k\}_{\mathcal{I}^k})$ Prior of model subset $\{\mathcal{M}^k\}_{\mathcal{I}^k}$

$p(v_{\mathcal{M}_i^k} \mid \mathcal{M}_i^k)$ Model-specific flux prior

$p(v_{\{\mathcal{M}^k\}_{\mathcal{K}}} \mid \mathcal{D})$ Model set-averaged flux posterior, averaged over the full model set $\{\mathcal{M}^k\}_{\mathcal{K}}$

$v_{\mathcal{M}}$, $v_{\mathcal{M}_i^k}$ Model-specific fluxes for model $\mathcal{M}$, $\mathcal{M}_i^k$

$v_{\{\mathcal{M}^k\}_{\mathcal{I}^k}}$ Model subset-averaged fluxes, averaged over all models in subset $\mathcal{M}_{\mathcal{I}^k}$

$v_{\{\mathcal{M}^k\}_{\mathcal{K}}}$ Model set-averaged fluxes, averaged over all models in $\{\mathcal{M}^k\}_{\mathcal{K}}$

$p(v_{\mathcal{M}} \mid \mathcal{D})$, $p(v_{\mathcal{M}_i^k} \mid \mathcal{D})$ Model-specific flux posterior

References

[1] Greg Ashton, Noam Bernstein, Johannes Buchner, Xi Chen, Gábor Csányi, Andrew Fowlie, Farhan Feroz, Matthew Griffiths, Will Handley, Michael Habeck, Edward Higson, Michael Hobson, Anthony Lasenby, David Parkinson, Livia B. Pártay, Matthew Pitkin, Doris Schneider, Joshua S. Speagle, Leah South, John Veitch, Philipp Wacker, David J. Wales, and David Yallup. Nested sampling for physical scientists. *Nature Reviews Methods Primers*, 2(1):39, 2022.

[2] Brendon J. Brewer. Inference for trans-dimensional Bayesian models with diffusive nested sampling. *arXiv [stat.CO]*, 1411.3921, 2015.

[3] Brendon J. Brewer and Daniel Foreman-Mackey. DNest4: Diffusive nested sampling in C++ and Python. *Journal of Statistical Software*, 86(7):1–33, 2018.

[4] Brendon J. Brewer, David Huijser, and Geraint F. Lewis. Trans-dimensional Bayesian inference for gravitational lens substructures. *Monthly Notices of the Royal Astronomical Society*, 455(2):1819–1829, 2015.

[5] Brendon J Brewer, Livia B Pártay, and Gábor Csányi. Diffusive nested sampling. *Statistics and Computing*, 21(4):649–656, 2011.

[6] Andrew Fowlie, Will Handley, and Liangliang Su. Nested sampling with plateaus. *Monthly Notices of the Royal Astronomical Society*, 503(1):1199–1205, May 2021.

[7] Zixiao Hu, Artem Baryshnikov, and William Handley. AEONS: approximating the end of nested sampling. *Monthly Notices of the Royal Astronomical Society*, 532(4):4035–4049, 2024.

[8] Johann F Jadebeck, Wolfgang Wiechert, and Katharina Nöh. Practical sampling of constraint-based models: Optimized thinning boosts CHRR performance. *PLOS Compututational Biology*, 19(8):e1011378, 2023.

[9] Johann Fredrik Jadebeck, Wolfgang Wiechert, and Katharina Nöh. Trans-dimensional diffusive nested sampling for metabolic network inference. *Physical Sciences Forum*, 12(1):5, 2025.

[10] Christopher P. Long and Maciek R. Antoniewicz. High-resolution ^{13}C metabolic flux analysis. *Nature Protocols*, 14(10):2856–2877, 2019.

[11] Doris Schittenhelm and Philipp Wacker. Nested sampling and likelihood plateaus. *arXiv [math.ST]:2005.08602*, 2021.

[12] John Skilling. Nested sampling for general Bayesian computation. *Bayesian Analysis*, 1(4):833 – 859, 2006.

[13] Axel Theorell and Katharina Nöh. Reversible jump MCMC for multi-model inference in metabolic flux analysis. *Bioinformatics*, 36(1):232–240, 2020.
